# Tumor Dormancy and Reactivation: The Role of Heat Shock Proteins

**DOI:** 10.3390/cells13131087

**Published:** 2024-06-23

**Authors:** Haneef Ahmed Amissah, Stephanie E. Combs, Maxim Shevtsov

**Affiliations:** 1 Institute of Life Sciences and Biomedicine, Department of Medical Biology and Medical Biology, FEFU Campus, Far Eastern Federal University, 690922 Vladivostok, Russia; haneefamissah@gmail.com; 2 Diagnostics Laboratory Department, Trauma and Specialist Hospital, CE-122-2486, Central Region, Winneba P.O. Box 326, Ghana; 3 Department of Radiation Oncology, Technische Universität München (TUM), Klinikum Rechts der Isar, 81675 Munich, Germany; stephanie.combs@tum.de; 4 Laboratory of Biomedical Nanotechnologies, Institute of Cytology of the Russian Academy of Sciences (RAS), 194064 Saint Petersburg, Russia; 5 Personalized Medicine Centre, Almazov National Medical Research Centre, 197341 Saint Petersburg, Russia

**Keywords:** dormant tumor, dormant tumor cell reactivation, dormant tumor cell, extracellular matrix, tumor microenvironment, heat shock proteins

## Abstract

Tumors are a heterogeneous group of cell masses originating in various organs or tissues. The cellular composition of the tumor cell mass interacts in an intricate manner, influenced by humoral, genetic, molecular, and tumor microenvironment cues that dictate tumor growth or suppression. As a result, tumors undergo a period of a dormant state before their clinically discernible stage, which surpasses the clinical dormancy threshold. Moreover, as a genetically imprinted strategy, early-seeder cells, a distinct population of tumor cells, break off to dock nearby or extravasate into blood vessels to secondary tissues, where they form disseminated solitary dormant tumor cells with reversible capacity. Among the various mechanisms underlying the dormant tumor mass and dormant tumor cell formation, heat shock proteins (HSPs) might play one of the most important roles in how the dormancy program plays out. It is known that numerous aberrant cellular processes, such as malignant transformation, cancer cell stemness, tumor invasion, metastasis, angiogenesis, and signaling pathway maintenance, are influenced by the HSPs. An accumulating body of knowledge suggests that HSPs may be involved in the angiogenic switch, immune editing, and extracellular matrix (ECM) remodeling cascades, crucial genetically imprinted strategies important to the tumor dormancy initiation and dormancy maintenance program. In this review, we highlight the biological events that orchestrate the dormancy state and the body of work that has been conducted on the dynamics of HSPs in a tumor mass, as well as tumor cell dormancy and reactivation. Additionally, we propose a conceptual framework that could possibly underlie dormant tumor reactivation in metastatic relapse.

## 1. Introduction

Tumors are a heterogeneous group of cell masses that arise in various organs or tissues, including the central nervous system [1,2]. These abnormal, heterogeneous cellular masses are composed of tumor cells, stromal cells, and immune cells that interact in an intricate manner and are modulated via the fluctuations in humoral, genetic, molecular, and tumor microenvironment cues that influence tumor growth or suppression.

Mounting evidence supports that, to this end, prior to the development of clinical symptomatology, primary tumor masses undergo a dormant state of balanced cell death and proliferation to maintain tumor size, events that are influenced by angiogenic and immune cascades [3]. Here, there is evolving crosstalk of pro-angiogenic and antiangiogenic factors that causes hypoxia in different parts of the tumor mass, drawing into play the unfolded protein response system, as well as a countering equilibrium between immune reactive and suppressive factors. Simultaneously, as a genetically imprinted strategy and evolutionarily conserved mechanism, early-seeder cells separate from the primary tumor site in the early stages of tumor development to dock nearby and/or enter blood circulation, where they are carried to distant organs and tissues as circulating tumor cells (CTCs). CTCs may survive the conditions in their new microenvironment, where they form what has become known as dormant tumor cells (DTCs). Characteristically, these dormant, solitary tumor cells are described by the hallmarks of proliferative reversibility, cell cycle arrest, resistance to radiochemotherapy, dependency on niche characteristics, a capability for metastatic relapse, and immune evasion [4]. As a result of their proliferative reversibility, dormant tumor cells can become reactivated at a later stage in the course of the tumor, constituting the residual disease seen in metastatic relapse [4].

Among the various mechanisms of dormant cell formation, heat shock proteins (HSPs) might play one of the most important roles. HSPs are a family (including small HSPs, HSP40/DNAJ, HSP60, HSP70, HSP90, and large HSPs) of highly conserved and ubiquitous proteins in both eukaryotic and prokaryotic organisms that are involved in the folding and unfolding of proteins and polypeptides (i.e., proteostasis), protein complex assembly, and cell protection from stresses (e.g., hypoxia, low pH, oxidative stress, ionizing radiation, etc.) [5]. It is not surprising, given the protective role of proteins, that HSP expression is significantly increased in many tumor types and correlates with the resistance of cancer cells to treatment methods, including radiochemotherapy [6,7,8,9,10]. It is worth noting that HSPs are also directly involved in many other mechanisms of tumorigenesis, such as invasion and metastasis, angiogenesis, EMT transition, the maintenance of signaling pathways, and other processes [11,12,13]. However, the role of chaperones in maintaining the dormant state of cancer cells is currently only beginning to be studied.

Globally, an incidence of 19.3 million tumor cases and a mortality rate of almost 10 million cases were reported in 2020 [14]. In 2024, 2,001,140 new cancer cases and 611,720 cancer deaths are projected to occur in the United States [15]. Even though the cases of tumors continue to rise globally, the dynamics of tumor dormancy and reactivation, a key factor accounting for tumor relapse, are poorly understood, particularly in brain tumors like glioblastomas that show a high relapse rate. In this review, we highlight the influence of HSPs on tumor dormancy initiation and reactivation, with special attention to brain tumors. Also, we propose a conceptual framework that possibly accounts for dormant tumor reactivation cascades, as reported clinically.

## 2. Concept and Biology of Tumor Dormancy

### 2.1. Concept

Tumor dormancy is an intricate process involving cellular, humoral, and molecular signals and tumor microenvironment interactions. It can be classified into tumor mass dormancy and cellular dormancy. Tumor mass dormancy represents a period of stagnation in tumor growth depicted by the balanced and synchronous tumor cell division and death in the micrometastases, which translates into a clinically indiscernible stage. The tumor mass steady-state is posited to arise from angiogenic and immune-mediated cascades [16], a state that is modulated in part by the dynamics of hypoxia, tumor microenvironment, autophagy, and genetic and epigenetic factors.

Tumor cell dormancy, on the other hand, refers to solitary tumor cells that exhibit reversibility into active proliferation after a period of growth cycle arrest (G_0_-to-G_1_ in the cell cycle). Dormant tumor cells arise from DTCs of extravasated early-seeder circulating tumor cells (CTCs). Apart from their growth reversibility capacity, dormant tumor cells have a decreased metabolic state and an altered chromatin and epigenetic structure at the molecular and sub-molecular levels, which facilitates their prolonged period of dormancy [17,18,19].

Equally crucial for the maintenance and sustenance of the dormant tumor cell’s extended period of dormancy is its niche. The niche in which the DTC cells are found is maintained through *Wnt*/*Notch* signaling [20], *TGFβ* [21], mitogen and stress-activated kinase 1 (*MSK1*) [22], Hedgehog proteins [23], and the bone morphogenetic protein (*BMP*) [24] pathways, a majority of which converge on the mammalian target of rapamycin (mTOR), mitogen-activated protein kinase (MAPK)/extracellular regulatory kinase (*ERK*), and the *p38* effector molecules [4,25]. To maintain the cell dormancy program, small GTPase *Cdc42* has been linked, as reported by Aguirre-Ghiso et al., to upregulating *p38* signaling and instigating growth arrest [26].

However, while dormant cells possess reversibility capability following long periods of dormancy, the capacity of senescent cells to exhibit the same remains an open discussion. Recently, Prunier et al. demonstrated that dormant breast cancer cells and not senescent cells depict growth reversibility after growth arrest with a tetraploid genome (G1-arrested tetraploids, 4NG1) that protects the cell from undergoing aneuploid changes [27]. While the phenotypical, genetic, and epigenetic signature of senescent cells is not well defined, and the possibility of their reversibility into active proliferation is debated, the tetraploid presentation of dormant cells opens a whole new chapter for further discourse and consensus.

### 2.2. Biology of Tumor Dormancy

**Figure 1:**
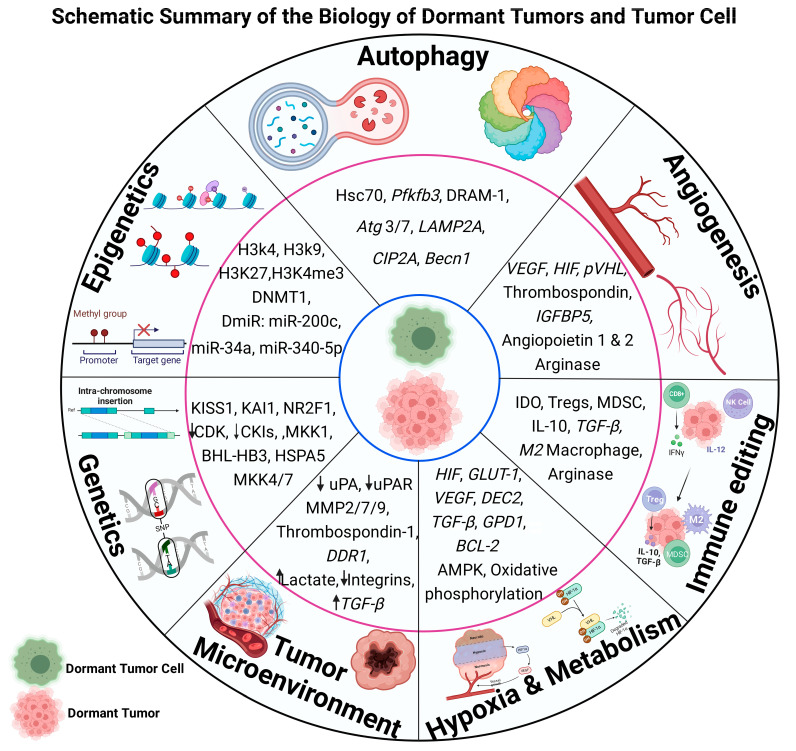
Summary of the biological processes and their related molecular, genetic, and pathophysiological mechanisms that drive and sustain the tumor dormancy and tumor cell dormancy program.

The dynamics of the tumor growth and cell dormancy landscape are a plethora of survival strategies: strategize and survive or relent and go extinct. To do this, tumor cells undergo molecular and genetic remodeling events that are crucial for survival in an unfriendly environment. Among these events are genetic and epigenetic changes, the remodeling of the tumor microenvironment, hypoxia, autophagy, immune editing, and angiogenesis to control and sustain the dormancy program. A concise summary of the biology of tumors and tumor cell dormancy is presented in Figure 1. In this section, we look at these developments and how they influence the dormancy process.

#### 2.2.1. Genetics and Epigenetics

Genetic and epigenetic events in the dormant tumor cell are pivotal regulators of the tumor dormancy program. Epigenetic and genetic events are intrinsic and extrinsic cellular responses that manifest in the upregulation and downregulation of genes, as reflected in the phenotypic plasticity of tumor dormancy across the flow of biological information: transcriptional, posttranscriptional, translational, and posttranslational stages. The execution of this program ensures precise control of the tumor dormancy program with proliferative reversibility as an indispensable core.

Hypoxia, a trait common to most tumors, plays a significant role in the setting of the tumor microenvironment tumor cells for inducing the expression of hypoxia and hypoxia-associated genes, some of which support tumor dormancy. Here, it has been observed that the *TET1* and *TET3* genes are upregulated in response to hypoxia [28]. In their study, Wu et al. highlighted the induction of stem-like traits in breast cancer cells through a putative *HIF-α* binding site in the promoter regions of the *TET1* and *TET3* genes [28]. These genetic regulation and expression events are associated with hypoxia factor *HIF-α* expression, which subsequently enhances *TNF-α*-driven *p38-MAPK* signaling and regulation to control cancer progression via the prior activation of the *TET* genes. The dynamics of these genetic expressions are a common feature reported in both breast and prostate malignancies.

Similarly, in a study by Sun et al., the authors observed that the chromatin-remodeler, high-mobility group AT-hook 2 (HMGA2) knockdown in breast cancer cells induced *TET1* gene expression. Subsequent to *TET1* gene expression, *TET1* auto-demethylates its promoter and, subsequently, the demethylation of the promoter of homeobox A (*HOXA*) genes, *HOXA7* and *HOXA9*, culminating in the effect from the *TET1*, *HOXA7,* and *HOXA9* genes suppressing breast cancer invasion and metastasis [29]. Consequently, *HOXA9* expression enhances *H3K4me3* demethylation, which culminates in the stemness of breast cancer cells by suppressing cellular differentiation [30]. The ultimate development of the stemness and reduced cellular invasion of breast cancer cells post the hypoxic conditions is further maintained via the expression of tissue inhibitors of the metalloproteinases (TIMP) family proteins 2 and 3 to sustain the dormancy features [31].

Other genes involved in the regulation of dormancy include the following: *KISS1* [32], *KAI1*/*CD82* [33], *MKK4/7* [34,35], and *NME/AKAP12* [36,37]. For example, *KISS1* interferes with cell migration and invasion by shifting the dormant cell’s metabolism from glucose and lipid metabolism to oxidative phosphorylation and β-oxidation [38]. Similarly, the T-box transcription factor-2 (*TBX2*) has been shown to bind and repress the *p21^WAFI^* cyclin-dependent kinase inhibitor gene promoter to arrest cell growth [39]. Also, *AKAP12*, via the *STAT3* and protein kinase C pathways, suppresses cancer cell colony and spheroid formation while maintaining stem cell-like phenotypes [40].

In a tissue-specific manner, the mitogen-activated protein kinase kinase 4/7 (*MKK4*/*7*) regulates tumor cell dormancy in metastatic DTCs, primarily through the mitogen-activated pathogen kinase (*MAPK*) pathways, the c-Jun N-terminal kinase (*JNK*)-mediated pathway in prostate cancers [34], and the *p38* pathway in ovarian cancer cells via cyclin D1 inhibition and *MAPKAPK2* and *p53* upregulation [41]. Additionally, *MKK4* can activate *p21* to drive CDK inhibition and retinoblastoma (*RB*) phosphorylation to arrest the cell cycle [42].

Furthermore, the mitogen and stress-activated protein kinase-1 (MSK1/MAPK1), a key player in tumor dormancy, is instrumental in regulating dormant cell stemness [19,22]. The MSK1 regulation of tumor dormancy via *p38* is associated with the basic helix-loop-helix family member e41 (*BHLHE41*/*DEC2*) [43], the orphan nuclear receptor 1 (*NR2F1*) [44,45], *BHLHB3* and *p53* [46], and the downregulation of c-Jun and forkhead box protein M1 (*FoxM1*) [46] through *p21* and *p27* induction to tighten the inhibition of the cyclin-CDK complex [47]. Also, epigenetic factors like histone H3 methylation and *H3K4*, *H3K9*, *H3K27*, and *NR2F1*-induced global chromatin-structure repression compact and maintain chromatin integrity during dormancy [48,49].

Coupled with the aforementioned procesess, cell survival genes like *HSPA5*, *DDIT3*, and *RPS6KB1* are upregulated in an autophagy-mediated mechanism [50], while the central nervous system presents a distinct genetic picture with *EphA5*, *IGFBP5*, and *H2BK* gene upregulation [45]. This variance in the genetic and epigenetic landscape could possibly account for the characteristic non-metastatic nature of most central nervous system tumors.

Epigenetic modifications like DNA methylation are crucial for eukaryotic genome regulation [51], and this is no different in dormant tumor cells but in a different dimension. Such modifications manifest as hyper- and hypomethylation processes, which result in diverse regulatory patterns of gene expression. For instance, DNA methyltransferase 1 (*DNMT1*) maintains tumor cell dormancy and stemness by blocking the G_1_/S phase transition signaling network [46]. The orphan nuclear receptor (*NR2F1*) regulates tumor cell dormancy in head and neck squamous cell carcinoma (HNSCC) by coordinating the hypermethylation of H3 histone proteins (*H3K27*, *H3K9*, and *H3K4*), bounded to *SOX9*, *RARβ*, and CDK inhibitors to inhibit cell proliferation [48]. Additionally, the *NR2F1* gene also induces chromatin repression, which results in the suppression of the pluripotency gene *NANOG*; blocking *NR2F1* reverses this process, consequently.

Genome-wide hypomethylation, primarily in gene-coding regions and satellite repeats, leads to chromosomal rearrangement and mitotic recombination, causing significant genomic instability that promotes phenotypic heterogeneity, as seen in tumors [52]. Furthermore, microRNAs (*miR*) are potent dormancy epigenetic players in what has become known as dormancy-associated *miR* (*DmiR*). For instance, *miR-34a*, *miR-93*, and *miR-200c* are epigenetic regulators of osteosarcoma dormancy; however, their loss is linked to angiogenic and proliferative switches driving metastatic relapses [53]. Also, *DmiRs miR-190*, *miR-580*, and *miR-588* promote dormancy switching and transcriptome regulation in angiogenic glioblastomas and osteosarcomas by downregulating pro-angiogenic factors and promoting anti-angiogenesis [54].

In the same vein, *DmiR* has been implicated in regulating tumor dormancy by regulating HSPs like the *DmiR miR-340-5p*, which suppresses hepatocellular carcinoma growth by blocking the cyclic AMP-dependent transcription factor-7 (*ATF7*), a target of the HSP *HSPA1B* that induces cellular proliferation [55,56]. Also, the DmiR miR202 induces esophageal squamous cell carcinoma (ESCC) dormancy by regulating heat shock transcription factor 2 (*HSF2*) and its target gene, HSP70, hence suppressing ESCC in a dormancy state, and vice versa [57]. miR202 inhibits *HSF2* by targeting the *HSF2* mRNA in the 3′UTR to induce apoptosis through caspase-3 activation. However, *HSF2*/HSP70 upregulation inhibits miRNA to promote cell survival in a dormancy-like manner.

Given the functions of cyclins CDKs, and CDKIs, it is essential for proper cell cycle and growth that their corresponding genes are correctly regulated. Thus, genes that promote dormancy, such as the CDKIs, have acetylated histones and phosphorylated genes for constitutive expression, while proliferation-inducing genes, such as cyclins, are methylated [58]. Also, in breast cancer, hypermethylation of the WNT ligand prevents Hedgehog pathway activation, downregulating the *DKK3* promoter and inhibiting *GSK3β* from degrading β-catenin [59]. The effects of these genes and epigenetic forces suppress tumor growth and metastasis.

However, as demonstrated via metastatic relapses, a much stronger force can subvert these genetic and epigenetic circuitry networks, overturning the dormancy program to ignite proliferation pipelines. Not to be overlooked is the role played by the tumor microenvironment in the reconfiguration cascades of tumor dormancy, which reorganize the genetic and epigenetic mechanisms that force tumor dormancy into active proliferation.

#### 2.2.2. Dormant Tumor Microenvironment

The cell microenvironment plays a crucial role in cellular fate and behavior [60], with tumor cell–ECM and tumor cell–stromal interactions determining progress or regression [61,62]. To corroborate tumor development, a supportive microenvironment is vital through microenvironment remodeling [63]. The possibility is that these developments ride on the ECM’s plasticity to precisely direct ligands to cell receptors in order to influence timely tumor progression or regressive modes.

To accomplish this, tumor cells contribute to ECM landscaping by secreting constituents that corroborate their behavior and tumor establishment. Hence, dormancy-promoting ECM biomolecules released in the dormant tumor cell’s microenvironment direct *p38* pathway activation in the early-stage tumor cell–ECM crosstalk [4,64], increasing *INK4* and *Cip*/*Kip* family proteins, CDKIs, and E2F transcriptional inhibition via the *MAPK*/*TGFb2* pathway to inhibit CDK4/6 and Cyclin-CDK complexes to arrest tumor cells in the G_0_-G_1_ phase [58].

The survival and sustenance of dormant tumor cells depend on maintaining their ECM composition. Other factors, such as metastatic niches, hypoxic microenvironments, and endoplasmic reticulum stress, also play a role [65,66]. Undeniably, cell–ECM interactions, cell–cell communications, and secreted factors, such as interferon [67], are crucial for dormancy.

Also, a great deal of evidence suggests that dormant cells adhere loosely to the extracellular matrix (ECM) [64,68,69,70], leading to low E-Cadherin and uPAR levels [71,72]. In a study by Correa et al., it was observed that floating spheroids from ovarian cancer patients expressed high levels of *p130*, an Rb-like protein, and a characteristic low *ERK*/*p38* ratio that promoted dormancy due to the loss of the cell–ECM interactions [73]. This sustains and promotes dormancy, allowing mutational events that promote drug-resistant phenotypes. For instance, dormant lung cancer cells exhibit drug-resistant phenotypes with a characteristic inhibition of the *ERK1*/*2* and *Akt* pathways that explain the downregulation of tumor progression and invasion genes (*uPA*, *uPAR*, *MMP2*, *MMP7*, *MMP9*, and *CXCR4*) [74]. 

Moreover, ECM remodeling alters the ligand and architectural structure, leading to nanomechanical changes. For example, β1-integrin suppression in breast cancer promotes dormancy by inhibiting actin stress fiber formation [21]. Further, HNSCC exhibits increased type III collagen levels that modulate the tumor cell dormancy cascades by inhibiting *DDR1*-mediated *STAT1* signaling [65,75]. Resultantly, these changes alter ECM stiffness, with soft ECM supporting dormancy and stiff ECM promoting proliferation [76,77,78,79]. Recent advances in decellularized matrices and bioengineered 3D matrices show reduced adhesivity and degradability for dormancy and survival, while increased adhesivity and less degradability increase cell predisposition for cellular reactivation, proliferation, and invasion [80,81,82,83].

Typically, the dormant tumor milieu is known for its acidic microenvironment due to uneven blood and lymphatic fluid distribution, which redirects cellular metabolic pathways to aerobic glycolysis with an increase in lactic acid [84,85] and the inhibition of the *Raf*/*ERK*/*mTORC1* pathway [86,87]. It also promotes stemness and stem-like cell markers in glioma cells in an *HIF-2α*-dependent, angiogenic-factor manner [88]. However, some reports have demonstrated differing observations of tumor proliferation and invasion, rather than the dormancy-promoting effect of tumor microenvironment acidosis [89,90]. 

Specific microenvironments require different signaling and dormancy induction systems. For example, by binding to annexin II within hematopoietic stem cell niches, which is triggered via osteoblast-derived growth arrest-specific 6 (*GAS6*), disseminated prostate cancer cells upregulate *Axl* receptors and *TGF-β* receptors, a process that is linked to cellular dormancy [91]. Remarkably, sprouting neovasculature tip niches show differential expression of the anti-angiogenic factors *TGF-β1* and periostin that promote tumor growth in bone marrow and lung cancers, in contrast to thrombospondin-1 (*TSP-1*) expression in the mature lung and bone marrow microvascular niches that supports dormancy through its anti-angiogenic capacity [92]. The dynamism of the intratissue growth and dormancy differences draws a sharp contrast to how the varied ligand composition of the ECM milieu plays out in the dormancy program.

Interestingly, the ECM biomolecular composition of dormant tumor cells and stem cells varies. Collagen IV and VI are crucial in dormant hematopoietic and muscle stem cell microenvironments [93], while neural stem cells in the brain’s subventricular zone are maintained in a niche rich in laminin, collagen, nidogen, and proteoglycan [94]. Speculatively, the key distinction between physiological stem cells and CSC lies in the derivation of tumor cells and tissues from CSC and the physiological stem cells’ ability to regenerate the hierarchical tiers of biological systems, but they both present a seemingly comparable ecosystem.

Also, the poorly vascularized tumor microenvironment creates a favorable haven that shields DTCs from therapy [95] and promotes immune editing and angiogenic dormancy processes.

#### 2.2.3. Angiogenesis

Angiogenesis is a tightly regulated and crucial process for optimal brain physiology. However, as a result of pathological angiogenesis that exploits the dysregulation of angiogenic and anti-angiogenic factors, brain tumors upregulate blood vessel formation for excessive tumor growth and progression [78], a rate-limiting step in the tumor growth program [96]. But solid tumors like brain tumors depict vessel abnormalities with disordered growth and distribution patterns [82,83,97,98]. Thus, equilibrium in the proliferation of tumor cells in vessel-enriched regions and cell deaths in vessel-deprived regions create tumor dormancy, as seen in some preclinical studies [99,100,101]. Dormant brain tumors like GBMs secrete high levels of thrombospondin, angiomotin, and insulin-like growth factor-binding protein 5 (*IGFBP5*) against a background of low endothelial-specific marker 1 (*ESM1*) and epithelial growth factor receptor (*EGFR*) to induce and sustain the tumor mass dormancy program [97,102] in a thrombospondin-mediated *PI3K* pathway [98,103].

The angiogenic process is ECM remodeling-dependent, potentiating the sprouting of activated endothelial cells into the perivascular niche. Resultantly, the angiogenic-mediated ECM remodeling actively restructures the perivascular niche stromal cell constitution that responds by releasing immune-modulating molecules. Hence, the tumor angiogenic dormancy process stimulates heightened endothelial, tumor cell, and stromal immune crosstalk requisite for driving immune dormancy. In this light, growing literature shows that the ECM components [101,102,104,105,106] influence a proinflammatory or immune suppressive response that influences tumor dormancy and the reactivation of dormant tumor cells.

#### 2.2.4. Immune Editing

Immune surveillance, a conserved evolutionary measure through innate and adaptive immunity, ensures checks and balances by interacting with tissues and cells to eliminate infected and abnormal cells. In that regard, tumor cells, as expected, are to be eliminated via the immune system. But, through immunoediting, stepwise crosstalk between the immune cells and tumors stimulates tumor immunogenicity mechanisms keen for tumor elimination, equilibrium, and/or escape [103,107]. Hence, the immunoediting cascades explain the possibility of dormancy and the aggressive recurrence of tumors in metastatic relapse despite the presence of a competent immune system.

Generally, the CD8+ cytotoxic T cells (CTL) and the CD4+ helper T1 (*Th1*) cells eliminate tumor cells via an interferon-γ (*IFN-γ*) and cytotoxin-mediated mechanism [105,108]. But early-stage T cell-mediated immunosurveillance instructions lead to increased *c-Myc* expression via a non-canonical *IFN-γ-STAT3* pathway that reengineers the bioenergetic program of tumor cells towards immune escape [106,109]. In the brain TME, astrocytes, and tumor-associated macrophages (TAMs) type 2 (*M2*) produce *IL-10* and transforming growth factor β (*TGF-β*), which inhibit the effector roles of TAM type 1 (*M1*) and CTL [110,111]. Specifically, gliomas produce indolamine 2,3-dioxygenase (*IDO*) that depletes tryptophan to shore up regulatory T cell (*Tregs*) levels while inhibiting CTL in the tumor milieu [112]. Note that the levels of amino acids are tightly regulated in the brain [107,112,113], and the high arginase levels produced via the M2 [110], microglia, and tumor-infiltrating myeloid cells counter T cell proliferation and their proper function [107,114].

Conversely, for the adult brain’s immune program, the transition from childhood through adolescence to adulthood is an immunosuppressive to an immunoresponsive shift [108]. Therefore, the immune dormancy landscape in pediatric brain tumors is relatively dissimilar from that of adult tumors. Thus, this area requires extensive inquisition to understand the subtle details. Even so, a study by Sandén et al. has shown that medulloblastomas present a unique blood humoral profile. That is, high vascular endothelial growth factor A (*VEGFA*) and *IL-7* and low *IL-17A* and *TNF-β* [109] could modulate immune dormancy and relapse.

Inherent to the metastatic relapse are additional mutations in the already unstable chromosomal structure of the dormant tumor cells that support immunoediting escape. Corroboratively, adult tumor cells portray extensive somatic mutations, while pediatric tumors accumulate a low mutational burden [26,87,113,114] but high epigenetic changes [111,115]. Molecular events that are driven by the preference for the error-prone, non-homologous repair system over homologous recombination cause genomic instability and mutational transformations [116,117].

These accumulated mutations in dormant tumor cells generate heterogeneous clones that evade immune surveillance. By extension, the increase in the tumor cells’ bioenergetics will cross the clinical threshold through mechanisms that are hastened due to mutational transformation.

#### 2.2.5. Hypoxia and Metabolic Dynamics

Most tumors are ladened with cellular hyperproliferation with poor vascularization that cause hypoxia. The physiological response is the induction of hypoxia-related genes (hypoxia-induced factor (*HIF*) and glucose transporter 1 (*GLUT1*)) and dormancy-modulating genes such as *NR2F1*, the basic helix–loop–helix family member e41 (*DEC2*), and the cyclic-dependent kinase inhibitor (*p27*) [118,119]. *HIF* is a heterodimer composed of two subunits: the cytoplasmic *HIF1-α* subunit translocates to bind the nuclear *HIF1-β* subunit to activate the hypoxia response element for hypoxia response gene transcription [120]. Notable are the pro-angiogenic factors vascular endothelial growth factor (*VEGF*) and fibroblast growth factor (*FGF*) that inhibit thrombospondin1 [121] and stimulate endothelial proliferation, leading to vascularization. The vascularization program sprouts tumor cell proliferation in the tumor mass.

However, the compressive and stiff tumor microenvironment disorganizes the vascularization process, resulting in the formation of leaky, dilated, and snaky blood vessels with a non-equidistant blood supply [122], causing unbalanced tumor cell growth and death, as seen in tumor mass dormancy. Juxtaposed to the DTCs in the metastatic niche, there is reversibility in the hypoxia-related gene expression that creates a low-oxygen milieu to promote chromosomal instability [119,123] while still maintaining dormancy, a phenomenon that supports the formation of the dormant tumor cell phenotypes [124,125].

Notably, Bragado et al. demonstrated that hypoxia induces *p38* signaling, which acts in conjunction with *NR2F1* and downstream *TGF-β2* signaling to influence the *ERK*/*p38* ratio in HNSCC to enforce the dormancy program [21] in a loop feedback system. The dynamics of this process, together with the reversibility of hypoxia-related pro-angiogenic gene expression, could contribute to the switch between a dormant cell or a proliferation-inducing system that can be explored via DTC in metastatic relapse later in the course of tumor growth, as exemplified in bone marrow metastatic breast cancer cells [126].

Notwithstanding the effect of hypoxia on the expression of pro-angiogenic genes and, ultimately, the induction of cell proliferation and the attendant metastatic relapse, the dormant tumor cell resorts to other strategies. DTC cells in the metastatic niche are known for their characteristic reduced metabolic state, a scenario that supports reduced metabolic activity in slow growth and growth arrest [127,128]. In relation to the altered cellular metabolism that is orchestrated by unfavorable metastatic microenvironment conditions, there is a shift towards oxidative phosphorylation, reactive oxygen species scavenging, and autophagy with reduced dependence on glucose metabolism [129,130]. Responsible for the modulation of the oxidative phosphorylation picture in dormant tumor cells is adenosine monophosphate-activated protein kinase (AMPK), driven by fatty acid oxidation in the mitochondria, a pathway that promotes anti-oxidative stress and, when inhibited, results in residual tumor elimination in preclinical studies [131]. The downstream effect of AMPK in sustaining the tumor cell dormancy program is the repression of the *mTOR* pathway [132].

Similarly, CSCs in solid tumors or DTCs with stem-like characteristics utilize oxidative phosphorylation [133], together with fermentative glycolysis [134]. The expression of glycerol-3-phosphate dehydrogenase 1 (*GPD1*) and the B-cell lymphoma 2 gene (*BCL-2*) is frequently observed in leukemia [135] and brain tumor cells [136,137,138] that are in a dormant state. These genes are involved in an effective oxidative phosphorylation program.

Aside from the oxidative phosphorylation pathway that sustains dormancy, dormant tumor cells also divert their metabolic intermediates towards glycerol and phospholipid metabolism [137,138] while also drawing in HSPs (autophagy) to ensure the prudent control and utilization of their scarce resources in a program whose end is not certain [139,140].

#### 2.2.6. Role of Autophagy

Autophagy is an evolutionarily conserved system that fosters homeostasis by causing the orderly recycling and degradation of cellular constituents. Cellular components to be recycled or degraded are transported as cargo in vesicles. Therefore, the autophagy system is divided into four categories, based on how cargo is delivered to lysosomes: macroautophagy, microautophagy, crinophagy, and chaperone-mediated autophagy [141,142].

The impact of autophagy on tumor establishment is tumor- and autophagy gene-specific. Notably, the autophagy pathway *Becn1* gene expression and the *Atg* family protein *Atg7* knockdown cause solely lung, liver, and lymphatic tissue tumor formation [143] and liver tumors [144], respectively, to depict organ- and tissue-specific effects of the autophagy system.

Moreover, the differential expression of the 6-phosphofructo-2-kinase/fructose-2,6-biphosphatase 3 (*Pfkfb3*) gene correlates with the induction of breast cancer stem cells’ (BCSCs) renewal, which fosters an aggressive breast cancer metastatic relapse, while its downregulation causes breast cancer dormancy. What is more, the targeted knockout of the *Atg* family proteins *Atg3*, *Atg7*, or p6/sequestosome-1 disrupts the autophagy system, which regulates the *Pfkfb3* gene, leading to the reactivation of dormant BCSC relapse [145]. Thus, *Pfkfb3* gene expression and the induction of the autophagy system are inversely related in expression, the dynamics of which are exploited in the tumor cell dormancy program. Further, Lu and colleagues remarked on the role of the Aplasia Ras Homolog member 1 (*ARHI*) in tumor dormancy [146]. As a result of *ARHI* re-expression in ovarian cancers, there was inhibition of the *PI3K/AKT/mTOR* pathway, which led to *Atg4* upregulation and its colocalization with cleaved microtubule-associated protein light chain 3 (LC3) in autophagosomes, allowing ovarian cancer cells to remain dormant. But *ARHI* downregulation in xenografts resulted in rapid tumor outgrowth and invasion, further underscoring the role of autophagy in tumor dormancy.

Similarly, the tumor suppressor gene *p53*, a master regulator of crucial cellular activities, is also implicated in the modulation of the autophagy system. In physiological states, *p53* represses autophagy [147]. But, in stress-induced states, a host of genes, including the p53-activated 5’-AMP-activated protein kinase subunit beta-1 (*PRKAB1*) and the damage-regulated autophagy modulator-1 (*DRAM1*), activate the autophagy pathways [148,149]. In a preclinical study, it was noted that the *Atg7* autophagy gene suppresses oxidative stress and *p53* activator *Nutlin-3*-mediated apoptosis while simultaneously delaying the onset of neurodegenerative diseases [150]. Deductively, the diversity in the autophagy system during stress inducts the chaperone-mediated autophagy system upon autophagy loss in response to the increased unfolded proteins to confer cytoprotective roles in stress states.

Through the selective degradation and repair of vital proteins, the chaperone-mediated autophagy (CMA) system helps stressed and nutrient-starved cells make the most use of their limited resources. Paradoxically, CMA appears to play an indirect role in the tumor dormancy regulation cascades. To illustrate, Han et al. demonstrated in their study [151] that CMA downregulation via the lysosomal protein *LAMP2A* knockdown in mainly metastatic carcinomas causes tumor mass dormancy both in vitro and in vivo by upregulating the *Atg5*-dependent macroautophagy pathway. Nonetheless, CMA upregulation promoted tumor growth and metastasis. Contrary to its passive role in tumor dormancy, CMA activity is pivotal in hematopoietic stem cells’ function and survival. As a result, CMA impairment disrupts proper protein quality control coordination and the requisite metabolic requirements for appropriate hematopoietic stem cell quiescence and metabolic function [152].

Intriguingly, CMA can also inhibit the malignant transformation of normal cells, specifically by regulating the quality control process of DNA repair. Here, following a genotoxic event, CMA acts by concurrently degrading the cell cycle checkpoint kinase 1 (*ChK1*) and stabilizing the Mre11-Rad50-Nbs1 (MRN) complex, hence ensuring DNA repair and the optimal progress of cell cycle events [153]. Correspondingly, given the versatile and multitasking role and regulation of the *MYC* gene and its gene products in cell-cycle regulation [154,155] and neoplastic transformation [154], it has drawn keen research interest. In this regard, Gomes et al. reported the role of the CMA system in orchestrating the ubiquitination and degradation of cellular MYC levels in fibroblasts because MYC contains two KFERQ-like motifs, respectively 267VEKRQ271 and 361VLERQ365 [156]. The regulation of cellular MYC levels occurs via the dephosphorylation of MYC at its Ser62, a process that is mediated by CMA indirectly via protein phosphatase 2 (*PPP2A*/*PP2A*) (*CIP2A*)-dependent inhibition to avert cancerous transformation in fibroblasts. Correspondingly, the inhibition of CMA led to an increase in cellular levels of *MYC* [156]. In support of the role of *CIP2A* in the *MYC* promotion of cancerous transformation, Puustinen and Jӓӓttela reported *CIP2A* as an oncoprotein that promotes *MYC*- and *MTORC1*-mediated cancer transformation by inhibiting the autophagy pathway [157].

Although autophagy, as reported here, supports a dormancy-like effect, it has a dual role, which is implicated in the promotion of cancerous transformations and tumor progression, as reported in other studies [158,159,160,161,162] and reviewed here [163].

Accordingly, tumor cell dormancy, as it is extensively studied in breast and bone malignancies, highlights the indisputable influence of the molecular components and characteristics of the niche ECM in regulating cell fate and behavior. But the characteristic dormant tumor cell niche of biophysical, biomechanical, and chaperone-mediated dynamics remains to be fully characterized and understood, particularly in tumors of the central nervous system. Furthermore, when considering the intensified demand for proteostasis, particularly in apoptotic regulation for extended periods of tumor cell dormancy, the chaperone-mediated autophagy pathway is an indispensable mechanism in this enterprise.

## 3. Heat Shock Proteins

HSPs are a ubiquitous, evolutionarily conserved family of proteins in eukaryotes and prokaryotes. The usefulness of HSPs is pronounced in cellular proteostasis and cytoprotection from various stress factors, including hyperthermia, hypoxia, cytotoxic agents, ionizing radiation, etc. Based on their molecular weights, HSPs are classified into two families: large HSPs (*HSP110*, *HSP90*, *HSP70*, and *HSP60*) that utilize ATP and small HSPs (*HSP40* and *HSP27*) that do not employ ATP [5,164].

HSPs are abundantly expressed in different compartments of the cell, with diverse functions depending on the physiological state of the cell. As molecular chaperones, HSPs participate in the folding of newly synthesized polypeptides, the refolding of metastable proteins, and orchestrating the degradation of misfolded, aggregated, and “worn-out” proteins via the activation of the ubiquitylation system, as well as serving as potent anti-apoptotic proteins [165]. To this end, in response to stressors, the cell releases hypoxia-inducible factor 1 (*HIF-1*) that binds directly to the hypoxia response elements (HREs) in the heat shock promoter to promote *HSF* expression [166] and induce chromatin remodeling for stress genes and HSP transcription [167]. In this regard, inhibitory feedback loop mechanisms in the *HSF1*/*HSP90* complex have been noted to regulate the transcriptional activities of *HSF1* [168,169]. Moreover, matrix metalloproteinase 3 (MMP-3) and heterochromatin protein 1 can also induce HSP transcription and activity [170]. Nevertheless, there is constitutive *HSF* and *HSP* expression even in the absence of stress that sustains oncoprotein folding, notwithstanding the “folding pressure” in cancer [171,172,173]. As a result, HSPs are increased in tumor cells and are also explored as a prognostic biomarker of disease progression and survival [174].

Although the functions of HSPs were proven to be intracellular, compelling evidence shows that some HSPs can be secreted extracellularly (eHSP) via free release from extracellular vesicles and microvesicles [175,176,177,178]. Notably, most ECM-remodeling enzymes depend on eHSP binding to ensure their stability and function. In the extracellular milieu, eHSP affects their functions by binding to and broadly activating the c-type lectin receptors (CTLR) and the scavenger receptors (SR), receptors with immune-modulating effects [179].

Notwithstanding their beneficial roles in cellular physiology, HSP dysregulation causes diseases such as cancer, neurodegeneration, cardiovascular diseases, and autoimmune and inflammatory conditions.

### 3.1. Heat Shock Protein in Tumor Dormancy

In order to satisfy the unplanned cellular activities of tumor cells to avoid apoptosis and safeguard their viability by inducing cellular dormancy, the dynamics of tumors and tumor cells require a balance in protein use and production. Even in this reduced cellular activity state of cellular dormancy, tumors still undergo mutational changes requisite to fortify cancer cells against anti-cancer agents, immunoediting, and preparing for metastatic relapse. To achieve the aforementioned traits, the remodeling of the ECM and cancer cells’ niche is key to achieving these feats, in which the HSPs are key players. Hence, it has become increasingly apparent that the tumorigenic process is acutely dependent on the combined mechanisms of protein folding executed via the molecular chaperones and their corresponding co-chaperones, which continue to be elucidated over time. Table 1 presents a summary of the roles of HSP in the tumor dormancy program.

#### 3.1.1. HSP in Tumor Angiogenesis Dormancy

Clinically discernible tumors become evident mostly in primary tumors after angiogenic sprouts that promote tumor cell proliferation, which increases the tumor size past Folkman’s visionary hypothesis (1–2 mm-size dormant tumors) in the reactivation drive of tumors [180].

Tumors and tumor cell dormancy are linked to the angiogenic switch and angiogenic dormancy, the latter of which reinforces dormancy. Angiogenic dormancy induces a vascular, poor TME that balances proliferation and apoptosis while instituting the cell-cycle arrest of the CSCs, or tumor stem-like cells, due to hypoxia and limited nutrient availability. In addition to *VEGF*, *HIF-1α*, angiopoietin 1 and 2, and basic fibroblast growth factor (*bFGF*), the main regulators of angiogenesis in health and in brain tumors are plasminogen activation inhibitor 1 (*PAI1*), nitric oxide, cyclooxygenase 2 (*COX2*), and thrombospondin 2 (*TSP2*) [181]. It is, however, practical to speculate that angiogenic dormancy inhibits or impairs tumor cell functioning, hence instigating the tumor cells to explore diverse metabolic routes aside from mainstream metabolism for survival.

Recapping the role of *HSP90* discloses its broad-ranging regulation of many oncogenic kinases and transcription factors, including *p53*, *HIF-1α*, *CDK4*, *BRAF*, *HER2*, *ERBB2*, *AKT*, *MEK*, *hTERT*, and *survivin* [182,183]; hence, an inhibition of HSP90 affects many physiological and tumor processes, including angiogenesis inhibition in many tumors [184,185].

In a study by Hadchity et al., the authors demonstrated the use of an antisense oligonucleotide, which knocks out *HSP27*, hence accentuating the effect of radiotherapy and improving the tumor prognosis in radiotherapy-resistant head and neck squamous carcinoma cells [186]. It was observed that *HSP27* knockdown was correlated with a reduced activation of the *Akt* pathway, which culminated in reduced angiogenesis. In a similar study, Straume and colleagues demonstrated that the downregulation of *HSP27* in breast cancers was correlated with a decreased expression of *VEGF-A*, *VEGF-C*, and fibroblast growth factor, hence knocking down angiogenesis to institute angiogenic dormancy [140]. Therefore, angiogenesis in breast cancer appears to be an *HSP27*-dependent process that is downregulated through inhibition. Hence, *HSP27*-level modulation could be a potential therapeutic target for breast and HNSCC.

The HSP dynamics in the tumor have been observed to play a part in the cascades leading up to angiogenic dormancy. The turnup of events is regulated in part through the regulation of the signaling networks of hypoxia-inducing factor (*HIF-1*) and the release of *survivin*, which is induced via *HSP70* and its co-chaperone, *Bag 3* [187]. Through *HSP90* binding and the activation of *Akt*, *survivin* is potentiated to inhibit apoptosis and secure CSC survival as a preclinical observation seen in melanoma, breast cancer, cervical cancer, colon cancer, and embryonic kidney cells. Correspondingly, *HSP90* has been reported as a potent stabilizer of *HIF-α* for angiogenesis in the normoxic environment [188] of the tumor mass juxtaposed with the hypoxic environment. Also, *HSP47*, as it is highly expressed in GBMs, is reported to modulate angiogenesis and TME remodeling through a *TGF-β*-mediated pathway, in addition to promoting glioblastoma stem-like survival [189]. By inducing stem-like characteristics, the glioblastoma cells were observed to have spheres, a characteristic that is highly linked to the stem cells. However, oxygen sensing and the attendant angiogenic dynamics are largely unexplored, to the best of our knowledge, in the dormant brain tumor microenvironment.

Furthermore, a study that characterized HSP based on the thematic hallmarks of cancers found a surge in the *TRAP1* (*HSP75*), *DNAJA3* (*TID1*), and *DNAJC19* (*HSP40 C19* member) (*HSP40* family proteins) genes across different cancers, proffering anti-apoptotic signals [190]. In a study by Bae et al., the authors explored the role of *TID1* in von Hippel–Lindau protein (*pVHL*)-mediated angiogenesis inhibition via *HIF-1α* ubiquitination and proteasomal degradation [191]. The team reported an enhanced interaction between *pVHL*-*HIF-1α* and downregulation of *VEGF* expression, owing to the enhanced interaction and stabilizing role of *TID1*, highlighting the notable role of *TID1* in anti-angiogenesis dynamics. However, *TRAP1* (*HSP75*), *DNAJA3* (*TID1*), and *DNAJC19* remain to be extensively explored in brain tumors regarding their roles in angiogenic dormancy. This provides a peeking window to ascertain the roles of these HSPs in angiogenic dormancy, from which further studies can be planned, based on preliminary developments.

On the whole, together with the above-mentioned HSP, the role of *TSP2* in the solitary dormant brain tumor cell’s microenvironment requires a reassessment, as these could provide promising insights into developing new therapies.

#### 3.1.2. HSP and Immune Modulation in Tumor Dormancy

Although the role of the HSP in immune-mediated dormancy is poorly understood, HSPs are thought to play a role in this process [170,192]. Collaboratively, immune cells express a repertoire of HSP-binding receptors such as toll-like receptors (*TLRs*), CD94 lectin-like natural killer receptor, *CD91*/*LRP1*/*A2MR* macroglobulin receptor, and scavenger receptor expressed by endothelial cells-1 (*SREC-1*) and required for T cell priming and the immune response [170]. Among these receptors, *CD91* binding and activation via eHSP transduce cancer-progressive downstream signaling. Initially, downstream signaling orchestrates immune and angiogenic dormancy, and later, the immune escape plan and reactivation of the dormant brain cancer cells lead to metastatic relapse and aggressive invasion.

Maintaining the immune cell repertoire in the tumor microenvironment is a crucial responsibility linked to *HSP90* in the immune-editing cascade. For that reason, it has been observed that *HSP90* inhibition is associated with the irreversible downregulation of critical cell surface antigens, costimulatory molecules, T cell αβ receptors, and activating receptors on natural killer cells (NK cells), including *CD3*, *CD4*, *CD8*; *CD28*, *CD40L*, *NKp30*, *NKp44*, *CD2, CD11a*, and *KARp50.3*, respectively [193]. The impact of this manifests in immune cell activation, proliferation, *IFN-γ* production defects, and other immunological activities indispensable to mounting and sustaining cytotoxicity against tumor cells. This prepares the immune system for the immune equilibrium phase and, subsequently, immune escape in the immune-editing program in tumor dormancy.

Equilibrium in immunoediting illustrates humoral levels of low anti-tumor (Interleukin-12 (*IL-12*), *IFN-γ*) cytokines compared with high tumor-promoting cytokines (*TGF*-β, *IL-10*, *IL-23*) [194,195,196,197,198]. This humoral dynamic relates to high *CD8+* T cells, NK cells, and *γδ*T cell levels juxtaposed with low natural killer T cells (NKT cells), *Foxp3+* Treg cells, and myeloid-derived suppressor cells (MDSCs) in a study that looked at the cellular constitution in the occult tumor milieu [199]. An in silico, multidimensional model with a spatially resolved, single-cell gene expression study of GBM shows that *IL-10*-releasing *HMOX1+* myeloid cells drive T cell exhaustion in the TME [197]. Consequently, the investigators isolated the T cell population in the GBM tumor samples and found a less proliferative HSP-expressing *CD8+* HSP *HSPA1A* subgroup and minimal proliferative *CD4+* HSP *HSPA1A* phenotypes that correlate with *IL-10* levels, supporting the hypothesis that anti-tumoral immunity in GBMs is relatively weak. However, the role of the stress-associated HSP *HSPA1A* subgroup in the tumor immune modulation process is not clearly defined. But the appearance of the stress-associated HSP *HSPA1A* subgroup preempts a possible role in these immune dynamics and requires extensive study to make the fine details clear.

The downstream effect of HSP on immunoediting and immune dormancy is not an all-supportive and progressive picture for achieving immune dormancy, and this is important to note. Remarkably, *HSP70* can promote NK cell activity in a *HSP70*/*Bag-4* surface-positive exosome-dependent manner for the immune elimination and cytotoxicity of pancreatic and colon cancer cells [200]. Moreover, in immunomodulation, *eHSP70* binds to MDSC via toll-like receptor 2 (*TLR2*) to stimulate *STAT3* activation and *IL-6* release for host defense in mouse and human studies [201]. These contrary scenarios present a sharp contrast, necessitating a context-based inquiry into immune dormancy in brain cancers and, even more importantly, in pediatric tumors, owing to their relatively naïve immunosuppressive dynamics.

Pediatric brain tumors are mainly characterized by low immunologic marker expression and a high immune regulatory picture of MDSCs and Treg cells [202,203]. These infantile immune dynamics support a preference for an early-phase equilibrium immunoediting process compared to a possible elimination seen in adult brain tumors. Moreover, the four different molecular subtypes of GBMs depict distinct immunological differences, warranting extensive study in this area to illuminate the shadowy side of HSPs in immune equilibrium that characterizes dormant tumors and cancer cells.

#### 3.1.3. HSP and ECM Client Proteins in Tumor and Cell Dormancy

The extracellular matrix (ECM) is a meshwork of non-cellular macromolecules that defines the spatiotemporal layout of organ tissues and provides structural support for optimal cellular function. The extracellular matrix (ECM) in the brain, for instance, is primarily composed of non-fibrous substances called lecticans and proteoglycans with specific lectin and hyaluronan-binding domains that potentiate the optimal function of the brain’s cellular makeup [204]. The predominant molecular constituents of the ECM at any given time instruct varying cellular behaviors, owing to the ECM’s plasticity.

While in the metastatic niche, early-seeder DTCs are known to crosstalk with the ECM [4], and there is a necessity for intermittent interactions with the ECM through a loose actin cytoskeletal network organization structure [80]. Correspondingly, the construction of a tumor dormancy-promoting and -sustaining ECM is an indispensable procedure that is under the regulation of the periodic action of ECM remodeling enzymes. Key among these ECM-remodeling agents are matrix metalloproteinases (MMPs), lysyl oxidase (LOX), heparanase, urokinase plasminogen activator receptor (uPAR), disintegrin and metalloproteinases with thrombospondin motifs (ADAMTs), and cathepsins, which are recognized as targets of the HSP [205].

As a key regulator of the TME-remodeling process, with a preference for collagens, aggrecan, elastin, and vitronectin, etc. [206], MMPs are regulated via eHSP. According to research on breast cancer, the eHSP complex, which constitutes Hop, HSP40, p23, HSP70, and eHSP90α, is essential for activating MMP2 for breast cancer invasion. However, without eHSP70, the complex becomes deficient in activating MMP2, with less motile and non-invasive phenotypes [207]. Further, by using a 3D collagen-1 assay to investigate mammary epithelial invasion and branching, Correia et al. discovered that cell invasion depends on extracellular HSP90β-dependent binding to the MMP3 hemopexin domain. As expected, blocking HSP90β decreased the mammary epithelial carcinoma invasion and branching, demonstrating its crucial function in stabilizing MMP3 during tissue microenvironment remodeling [208].

Furthermore, urokinase plasminogen activator (uPA) catalyzes the conversion of plasminogen into plasmin, hence activating uPAR, an essential regulator of tissue remodeling [209]. Despite playing a critical physiological role in wound healing, it has been shown to be dysregulated in pathological processes. Consequently, uPAR dynamics have prompted an inquiry into its tissue microenvironment functions, such as studying the relationship between uPAR and the HSPs, HSP70, and *MRJ* (*DNAJB6*). In a study by Lin et al., vitronectin adhesion and the activation of the MAPK pathway enabled the migration and invasion of renal and colorectal cancer. The subtleties of these downstream effects are tied to HSP70/*MRJ* (*DNAJB6*)/uPAR complex formation and function [210]. Unsurprisingly, uPAR-mediated cell–vitronectin adhesion and MMP2/9 downregulation were inhibited through the *shRNA* suppression of HSP70, MRJ, or both. This results in a restriction of cell growth and motility in a dormancy-like fashion.

Moreover, a preclinical study used *miR-29b* to target-block the 3′-UTR of both protein mRNA products of a LOX/HSP47 complex, a pivotal regulator of the covalent cross-link formation in collagen fibrils and the maturation of the ECM [211,212]. Given that the LOX/HSP47 complex was disrupted through the suppression of HSP47 and LOX products, an aberrant collagen-structured ECM meshwork structure was seen upon microscopic examination [211]. Alternatively, *CCl4*-treated mouse hepatic stellate cells had a typical ECM structure with decreased *miR-29b* expression, depicting a sharp contrast and the crucial role of the LOX/HSP47 complex in ECM remodeling and architectural layout.

Additionally, heparanase has been recognized for its role in ECM turnover processes. In this endeavor, Nobuhisa and colleagues followed the microenvironmental compositions that drive cell differentiation. They attributed the nuclear shuttling role of HSP90 to an elevated expression of heparanase that regulated cell differentiation [213]. As anticipated, the inhibition of HSP90 resulted in reduced cell differentiation, highlighting the significance of the nuclear translocation mechanism. Additionally, pro-ADAMTS9, an ADAMTS precursor protein that catalyzes the cleavage of versican, is partly regulated by the HSPs *gp96*/*GRP94*, *GRP78*, and *ERdj3* [214,215]. The SiRNA targeting of *gp96*/*GRP94* was noted to downregulate pro-ADAMTS9.

These enzymatic cascades help formulate the stromal milieu required for the tumor mass and cell dormancy program. In this endeavor, the HSPs—in particular, the eHSPs—have been found to be quite crucial in coordinating the survival of tumor cells. Nonetheless, investigations into the crosstalk between HSP and cathepsin are lacking, and an inquiry into this area may yield insights into the dynamics of tumor dormancy and reactivation strategies.

**Table 1:** HSPs in tumor and tumor cell dormancy.

HSP Family	Member	Location	Function	Reference
Angiogenic Dormancy				
HSP27	HSP27	Intracellular	Levels of HSP27 correlate with VEGF (VEGF subtypes) expression and angiogenic events. Low levels are seen in dormancy.	[140]
HSP40	HSP47	Intracellular	Modulates angiogenesis and TME remodeling through a *TGF-β*-mediated pathway while ensuring stem-like cell survival.	[189]
HSP70	*Tid1* (co-chaperone)	Intracellular	Enhances *pVHL*-dependent *HIF-1α* stabilization or ubiquitination to block *VEGF* expression or inhibition.	[191]
	*Bag 3* (co-chaperone)	Intracellular	Regulates angiogenesis by controlling *VEGF* expression and the release of *survivin* via the additive effect of HSP90 binding to sustain tumor cell survival in dormancy.	[187]
HSP90	HSP90	Intracellular	*HSP90* regulates many oncogenic kinases and genes, including *p53*, *HIF-1α*, and *survivin*, to ensure survival while maintaining angiogenic dormancy.	[182,183]
Complex	*TRAP1* (*HSP75–HSP70* family) *DNAJA3* (*Tid1–HSP70* co-chaperone) *DNAJC19* (*HSP40 C19* member)	Intracellular	An upregulation of *TRAP1*, DNAJA3, and *DNAJC19* genes is observed across different cancers, offering anti-apoptotic signals to maintain and sustain tumor dormancy.	[190]
Immune Dormancy				
HSP70	*HSP70*/*Bag-4*	Extracellular	Promotes NK cell activity in an *HSP70*/*Bag-4* surface-positive exosome-dependent manner for the immune elimination and cytotoxicity of cancer cells.	[95]
	*eHSP70*	Extracellular	Promotes the immunomodulating role of MDSC via toll-like receptor 2 (*TLR2*) to stimulate *STAT3* activation and *IL-6* release.	[201]
HSP90	HSP90	Extracellular	HSP90 is associated with the activation of T cell αβ receptors, and activating receptors on NK cells are crucial for proper immune cell priming and effector roles. Downregulation leads to immune dormancy.	[193]
HSP Client Proteins				
HSP40	HSP47/LOX complex	Extracellular	The LOX/HSP47 complex is crucial for the structural patterning of the ECM. Downregulation of HSP47 expressions leads to aberrant ECM structure and layout.	[211,212]
HSP70	HSP70/ *DNAJB6* complex	Extracellular	The HSP70/*MRJ*(*DNAJB6*) complex regulates urokinase-type plasminogen activator (uPA) and urokinase-type plasminogen activator receptor (uPAR), which are downregulated in tumor dormancy.	[210]
HSP90	eHSP90β	Extracellular	eHSP90β-dependent binding to the MMP3 hemopexin domain for MMP3 activation for TME remodeling. eHSP90β downregulation leads to tumor dormancy.	[208]
	HSP90	Intracellular	Nuclear shuttling of HSP90 controls heparanase functioning and influences cell differences that shift tumor dormancy.	[213]
	*gp96*/*GRP94* (HSP90 paralog)	Extracellular	*GRP94* catalyzes the activation of the ADAMTS precursor, pro-ADAMTS9, for the cleavage of versican and TME remodeling that supports dormancy.	[215]
Complex	Hop HSP40 p23 HSP70 eHSP90α	Extracellular	Hop, HSP40, p23, HSP70, and eHSP90α complex is essential for MMP2 activation and keen for the TME remodeling of collagen type IV, aggrecan, elastin, and vitronectin.	[207,216]

#### 3.1.4. HSP and Tumor Mass and Cell Resistance to Programmed Cell Death

Due to their cytoprotective role, HSPs are significantly expressed in tumors. Epichaperomes, molecular network complexes of chaperones and co-chaperones, have been found to be key pathological regulators of proteome-wide dysfunctions in cancers, including brain cancers [217]. Demonstrated as potent enhancers of tumor and cellular survival in cellular stress [218], the upregulation of the epichaperome genes *HSP90AA1*, *HSPH1*, and *HSPA8* promotes the equilibrium of radio- and chemoresistance seen in tumor and indirect cell dormancy [219]. Especially, there is a proportionate association between epichaperome expression levels and the tumor’s susceptibility to tumor-disintegrating agents [220]. In addition, by bypassing the programmed cell death role of *p53*, both Hsc70 and HSP90 can bind to mutant *p53* alleles to inhibit wild-type alleles [221,222]. Moreover, by binding cytochrome c and inhibiting apoptosome formation, HSP27 can control the extrinsic program cell death pathway to ensure cell survival [223,224], salvaging tumor cell death in dormancy.

Replicative senescence, an integral hallmark of tumors, ensures that cell survival is circumvented via telomere lengthening through telomerase. The elevated HSP levels seen in tumors influence this process. Notably, HSP90 [225,226,227], HSP27 [227,228], and HSP70 [226,229] are central. Also, HSP90 can chaperone telomerase to prevent telomere attrition [230], as well as stabilize the catalytic subunit of telomerase to confer tumor cell survival [231].

Furthermore, to endure the extended periods of lowered cellular activity, dormant cancer cells resort to enhancing their resistance to chemotherapy and radiation. It is, however, interesting to know that cellular quiescence in GBMs promotes tumor and cancer cell progression [232].

Although the role of HSPs in brain tumor and cell dormancy continues to gather momentum, it can be speculated that an increase in HSP27, HSP70, and HSP90 largely promotes cancer cell survival to escape apoptosis in tumor and cellular dormancy.

## 4. Dormant Tumor and Tumor Cell Reactivation

The reactivation of dormant tumors and cells is crucial for tumor relapse and metastasis with aggressive and drug-resistant phenotypes. After the surgical excision of the primary tumor, dormant tumor cells undergo rewiring and reprogramming, requiring circuitry rewiring and reprogramming.

Apart from angiogenic factors, vascular perfusion, and paracrine signaling via co-resident niche cells [233], fibrotic and inflammatory cascades, including inflammation, also reinforce the reactivation program [234]. Albrengues et al. demonstrated the release of neutrophil elastase and MMP-9 from neutrophil extracellular traps (NETs) during inflammation that modulates ECM remodeling [235]. These NET proteases orchestrate laminin cleavage to activate dormant tumor cells via an *α3β1*-dependent integrin signaling pathway. A phenomenon that illustrates immune dormancy is “threshold crossing” for immune escape. Moreover, a similar manner has been proposed to occur in vascular perfusion, toppling angiogenic dormancy to reactivate dormant tumor cells.

However, the success of the reactivation program depends on the fitness and readiness of the dormant tumor and tumor cells at any given time. The reactivation of dormant tumor cells requires chromatin and molecular modifications, a genetically imprinted evolutionary strategy for dormant tumors and cancer to withstand extinction.

### 4.1. Heat Shock Proteins in Dormant Tumors and Tumor Cell Reactivation

HSPs play a key role in dormant tumor cell reactivation, a role that has been thoroughly researched in HSP27, HSP70, and HSP90, as indicated in Table 2. For instance, eHSP90 is known for its role in ECM remodeling by interacting with MMP2/9, LOXL2, tissue-type plasminogen (tPA), and fibronectin to promote ECM restructuring [236]. Moreover, Gopal et al., in a study, underscored the role of eHSP90 in glioblastoma tumor growth and invasion [237]. The authors observed that the interaction between the eHSP90α-specific component and its client protein *LRP1* affects tumor cell motility by altering the polarity of the cell through the development of lamellipodia. The eHSP90-LRP1 interaction also regulates the immune response and tissue remodeling to override immunological and angiogenic dormancy and stimulate tumor growth.

Additionally, when co-administered with eHSP90 potentiator clusterin, it has been demonstrated in a preclinical study by Tian et al. to synergistically accelerate epithelial-to-mesenchymal transition (*EMT*) and increase breast cancer metastasis [238]. In a similar manner, eHSP27 also interacts with MMP-2/MMP-9 in a synergistic way to promote EMT-like processes and ECM remodeling in vitro and in vivo in GBMs [239]. Again, eHSP27 mediates angiogenesis by increasing *VEGF*-mediated cell migration through toll-like receptor 3 (*TLR3*) by activating *NF-κB* [240], an angiogenic dormancy threshold-crossing strategy. In neuroblastomas, it is observed that HSP60 confers pro-survival and pro-proliferative activity while promoting angiogenesis for the dormant tumor mass and cell reactivation and transformation [241].

Among its numerous roles, HSP also promotes ECM component biosynthesis, which influences the fate of dormant tumor cells. Here, HSP47, a collagen-specific molecular chaperone with mesostable monomer and/or hyperstable trimer domains, is essential for proper collagen peptide folding [242], an event that influences collagen availability for crosslinking. Also, HSP47 can stimulate glioma stem cell (GSC) stemness survival by upregulating angiogenesis and ECM restructuring genes (*CD44*, *LAMC1m*, *Col4A2*, *ITGB1*, *FN1*, and *MMP9*) for GSC reactivation, migration, and invasion through a *TGF-β* and *CD44* signaling pathway [189]. Nonetheless, HSP47 shRNA knockdown inhibits tumor growth, migration, and invasion, as reported by Zhao and colleagues [242].

Typically, reactivated tumor stem cells present chemo- and radiotherapy-resistant phenotypes. Tumors like breast cancers, ovarian cancers, leukemia, head and neck cancers, and esophageal cancers show shorter disease-free survival and chemotherapy resistance with a surge in HSP27. But unlike HSP27, HSP70 shows a correlative and predictive pattern to chemo- and radiotherapy in breast and lung cancers [243], as well as glioblastomas [244]. Comparatively, HSP90-mediated signaling has emerged as the chief modulator of radiotherapy resistance [245,246], as has been demonstrated in studies by inhibiting HSP90 activity [247,248]. Again, HSP90 can stimulate chemoresistance directly and indirectly to protect the tumor cells from the effects of chemotherapy that is initiated in metastatic relapse [249].

Moreover, therapy resistance in the area of immunotherapy has not been left out either. In CSCs and cancer-initiating cells, a concomitant administration of a specific HSP40 subfamily protein, *DnaJB8*-cytotoxic T lymphocyte-mediated immune escape, is used in the immune-editing program to promote tumor recurrence, maintain tumors, and support metastasis [250]. Similarly, in preclinical studies, the administration of human recombinant granzyme B in a perforin-independent manner to target HSP70-positive, undifferentiated colon cancer cells and 3D tumor spheroids induced caspase-3-mediated apoptosis in the tumor cells [251]. Additionally, HSP90 inhibition with an immunotherapeutic agent, ganetesip, enhanced the cytotoxic T cell killing of human-derived melanoma cells by upregulating the expression of interferon genes and potentiating the effect of anti-*CTLA4* and anti-*PD-1* therapies [252].

Furthermore, HSP90 exhibits a positive correlation with HSP 70 [253] and enhances the effects of the actin cytoskeleton, metalloproteinases, and metastasis-promoting proteins to protect CSCs from dying. Moreover, for pediatric brain tumors such as low-grade gliomas, glioneuronal tumors, pilocytic astrocytomas, embryonal tumors, and medulloblastomas, HSP90α is also linked to the reactivation of dormant brain cancer cells [254]. In addition to the constitutive expression of Hsc70 (*HSPA8*) through *FAK* and *Src* phosphorylation, it is also pivotal to dormant glioma cell reactivation to migration and invasion [255]. Nonetheless, the inhibition of HSP90 and its downstream *FAK*-mediated signaling pathway disrupts the dormant brain cancer cell reactivation program.

Notwithstanding the wealth of information and concepts on dormant brain cancer reactivation, a great deal of work still lies ahead in understanding the cascades of events that reactivate the dormant brain cancer cell in relapse. A longitudinal study of animal models with a longer lifespan will be a promising start due to the short lifespan of mice, noting that dormant cancer cell reactivity can take years.

**Table 2:** HSPs in dormant tumor and tumor cell reactivation.

HSP Family	Member	Location	Function	Reference
HSP27	eHSP27	Extracellular	Interacts with MMP-2/MMP-9 synergistically to promote the EMT-like process and ECM remodeling. Promotes angiogenesis via *VEGF*-mediated cell migration through Toll-like receptor 3 (*TLR3*) and *NF-κB* activation.	[239,240]
HSP40	HSP47	Intracellular	Essential for proper collagen peptide folding and packaging. Stimulates GSC stemness, upregulates ECM genes, and induces angiogenesis.	[189,256]
HSP60	HSP60	Intracellular	Promotes angiogenesis for the dormant tumor mass and cell reactivation and transformation.	[241]
HSP70	HSP70	Intracellular	Maintains tumor cell survival by mediating apoptosis in tumor cells. Confers chemo- and radiotherapy resistance. Orchestrates dormant glioma cell reactivation.	[68,243,244,254]
HSP90	eHSP90α	Extracellular	Influences dormant tumor cell polarity for lamellipodia formation for cell motility via an eHSP90α–*LRP1* interaction. Confers an immune modulatory and protective effect on tumor cells by regulating the expression of immune suppressive factors.	[252,257]
	HSP90α	Intracellular	Acts synergistically with HSP70 to promote actin cytoskeleton polymerization, metalloproteinase activation, and metastasis-promoting proteins to protect CSCs from brain tumor cell reactivation.	[249]
	Clusterin (co-chaperone)	Intracellular	Induces epithelial-to-mesenchymal transition (EMT) via *ERK* and *Slug* activation.	[258]

### 4.2. Extracellular Matrix in Dormant Tumor and Tumor Cell Reactivation

Dormant tumor cells, while still in their dormant state, interact sparingly with the ECM with a loose actin cytoskeletal network organization structure [68,69]. Unsurprisingly, the transition of dormant tumor cells to activate proliferative phenotypes comes with strong ECM interactions. To facilitate the transition process, ECM remodeling creates the needed ligands for tumor cell priming into proliferation, migration, and ultimately invasion.

Tumor cell reactivation culminates in tumor recurrence. Notable amongst the ECM changes that drive brain tumor recurrence is increased collagen and fibronectin, as indicated in Table 3. ECM components are abundantly low in the brain but highly expressed in other tissues. In a pediatric brain tumor recurrence study by Chen et al., the authors found an increase in collagen and a significant overlap in results between recurrent pediatric and adult brain tumors [259]. A recent study by Di Martino et al. discovered the key role of collagen type III in promoting tumor cell dormancy by activating downstream signals that promote dormancy via the noncanonical discoidin domain receptor 1 (DDR1). But the disruption of collagen type III blocked the downstream inhibitory signals that sustain dormancy, hence leading to the reactivation of the dormant tumor cell [75]. Considering that collagen and fibronectin metabolisms are intertwined, an experimental model hinged on collagen III and fibronectin will not be far-fetched to shed more light on the dormant brain tumor cell milieu.

Furthermore, fibronectin, MMPs, uPA, and uPAR have been found to be upregulated in tumor cell reactivation [260,261]. The uPAR stimulates fibronectin fibril formation in the tumor milieu and transduces *ERK* signals via *α5β1* integrin ligands to revive the dormant tumor cells into proliferation [262]. But uPAR downregulation inhibits fibronectin fibril formation, causing a low ERK-to-p38 ratio signal that arrests tumor cells in a dormant state [263]. Correspondingly, Chen et al. observed a proportional increase in fibronectin expression in high-grade glioma [79], an observation that drives tumor recurrence, validating fibronectin as a poor prognostic marker [264,265]. Evidently, a model based on fibronectin signaling in the reactivation program will shed light on the revival of the dormant brain tumor cell in metastatic relapse.

Moreover, comparative proteomic profiling of GBM and medulloblastoma revealed a distinct ECM picture in medulloblastoma. Trombetta-Lima and colleagues, who conducted the study, noted an increase in the dense fibrillary ECM proteins fibrillins and lumicans in medulloblastoma, which were not observed in GBM aside from collagen, that was seen in both tumors [266]. Additionally, recurrent medulloblastomas show increased leptomeningeal dissemination (LMD), dependent on vitronectin-ABL (Abelson) kinase binding that polymerizes actin filaments for *c-Myc* expression [267].

Similarly, recurrent GBMs show an upregulation of the *GALNT13*, *ROBO1*, and *ANTRXN1* genes [268]. These genes are key to ECM restructuring that induces mesenchymal subtype development and cell–cell adhesion. Therefore, the reactivated dormant GBM cells engage in ECM reorganization [269]. This reinforces the role of ECM in the brain tumor cell reactivation program. Particularly, the transduction of cues induces EMT activation for increased tumor cell motility and invasion, as seen in metastatic relapses. It will be interesting to investigate the predilection for the mesenchymal subtype in the reactivation phase. Certainly, there could be a peculiar ECM signature that accounts for this glioma subtype upon reactivation.

Also, brain tumor reactivation and metastatic relapse are characterized by increased chondroitin-sulfate proteoglycan glycosaminoglycans. Prominent among these glycoproteins that corroborate the phase shift are chondroitin sulfate (*CSPG4*/*NG2*), the protein tyrosine phosphatase receptor (*PTPRZ1*), and the CD44 receptor expression [270]. Moreover, the tumor cells secrete, in addition to the above Tenascin-C, which together increases the ECM glycocalyx bulkiness and ECM-integrin interactions for tumor cell migration [271].

Consequently, ECM remodeling proportionally increases ECM stiffness (Young’s modulus) [272] and also creates force-mediated remodeling due to the cell density in the increasing-sized brain tumor. In addition, the humoral dynamics in the tumor mass and cell milieu also contribute to ECM remodeling via *TGF-β*-induced LOX and the cytokine stimulation of MMPs [273]. Furthermore, a study by Acharekar et al. demonstrated the upregulation of collagen and the ECM proteases MMP2, MMP9, and PLEKHA7 in recurrent GBM patients [274]. Hence, the early phase of reactivation is a transient cell–ECM adhesion in an epithelial-like manner before the EMT-mediated cell migration and invasion later on in tumor progression.

Unquestionably, tumor cell reactivation and progression are driven by ECM ligand constituents that promote tumor cell migration. The supportive ECM ligand formulation occurs through sequential secretion, degradation, and restructuring. Therefore, the importance of understanding the biomolecular and nanomechanical properties and the chain of events that influence the dormant and reactivated tumor cell–ECM microenvironment cannot be overestimated. The successful characterization holds promise for designing targeted therapies and repurposing existing therapies for personalized patient treatment.

**Table 3:** ECM in dormant tumor and tumor cell reactivation.

ECM Family	Member	Function	Reference
Fibrous protein	Collagen	Increases tissue stiffness and elasticity to increase tumor cell migration and invasion via integrins. Increased in pediatric and adult brain tumors.	[259]
Glycoprotein	Fibronectin	Increased concurrently with collagens to increase tissue desmoplasia for increased tumor cell adhesion, migration, and invasion.	[263,264,265]
Glycoprotein	Fibrillin	Forms microfibrils that serve as a scaffold for elastin deposition, increasing tissue elasticity. Increased together with lumicans, specifically in medulloblastomas.	[266]
Keratan sulfate proteoglycan	Lumican	Increases fibrillar collagen crosslinking to increase tumor cell migration. Upregulated specifically in medulloblastomas.	[266]
Glycosaminoglycan	Chondroitin-sulfate proteoglycan	Pivotal in neural and glial scar formation, enhances CSC survival, reduces immune proinflammation, and enhances immune cell clearance.	[270]
Glycoprotein	Tenascin-C	Promotes angiogenesis and increases ECM glycocalyx bulkiness for enhanced cell/integrin-ECM interactions.	[271,275]

## 5. A Conceptual Circuitry Framework Regulating Tumor Reactivation

Enormous efforts towards understanding tumor dynamics continue to inch us closer to even deeper insights about cancer. A lot more questions still remain unanswered, for instance, the orderly sequence of molecular events that guide the dormancy induction program. What orchestrates the reactivation of dormant tumor cells?

**Figure 2:**
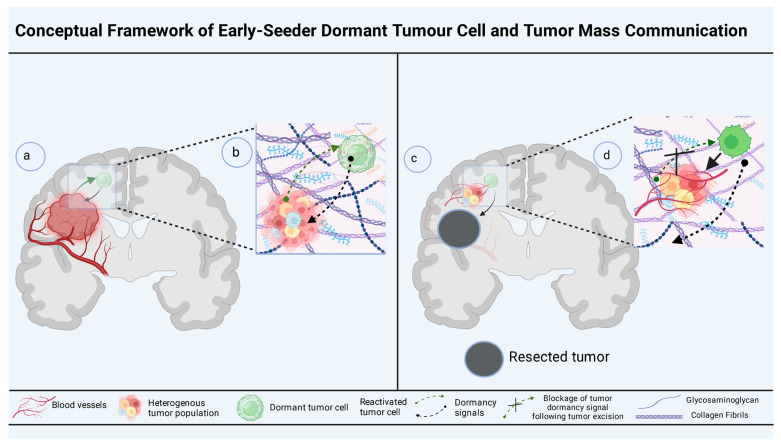
A schematic concept of the signaling mechanisms underlying dormant tumor relapse. (**a**) A brain tumor with a dormant tumor cell in the supratentorial brain region. (**b**) “Dormancy signals” exchange that takes place between the primary tumor and the dormant tumor cell to maintain the dormant tumor cells in their state of dormancy. (**c**,**d**) Cessation of communication between the dormant tumor cell and the primary tumor following the resection of the primary tumor cells that induces the dormant tumor cells into proliferation, migration, and invasion.

We, therefore, propose a concept that may possibly underline the relapse of dormant tumors long after the primary tumor has been resected, as illustrated in Figure 2 below. It is our view that, following the resection of primary tumors, the dormant tumor cells lose “signals” they receive from the primary tumor (“pheromonal-like signaling”) and resort to proliferating to ensure survival, a key evolutionary survival strategy. Moreover, could “quorum sensing” account for the reactivation and proliferative outburst that characterizes metastatic relapse?

## 6. Clinical Implications of Tumor Dormancy and Reactivation

The clinical implications of tumor dormancy lie in its unpredictable impact on the course of disease recurrence, progression, and treatment outcomes, all of which have an impact on the quality of life of patients, their families, and caregivers. Due to the dynamics of the dormant tumor mass and the biology of tumor cells, tumor cells can have the potential to reactivate and metastasize to distant organs and tissues, hence constituting residual disease even after the pronouncement of remission [276,277]. Even more troubling is are resistance and refractory nature of reactivated dormant tumor cells to conventional chemoradiotherapy, which stems from the high genetic instability, high mutational capability, and heterogeneous nature of tumors with unique intra- and intertumoral mutational profiles [278]. Therefore, tumor resistance restricts the options and scope for possible treatments with conventional therapies and is a contributing factor to treatment failure. Unexpectedly, fluorouracil, a component of many cancer drugs, has been shown to enrich dormant tumor cell formation [279].

Consequently, it has become more challenging to decide whether or not to treat dormant tumors or tumor cells, and if so, at what stage this can be initiated [280]. Since reactivated tumor cells or metastatic relapse present therapy-refractory phenotypes [281], the other puzzle is which combination and/or treatment plan to employ.

The main paradigms in these circumstances have been to eradicate the dormant cell population [282] or sustain the dormancy state to prevent reactivation [48]. A stumbling block in these strategies is the shut-down of the cell’s replication machinery, which is instrumental in the design mechanism of “cidal” and static-inducing medications—strategies that do not curb the reactivation of the dormant tumor cell.

Predictive and prognostic markers and tools for risk stratification look more promising and tangible in a deterministic strategy for clinical decision-making. Moreover, alternative therapies, such as immunotherapy, have shown some successes and failures. The search for novel therapies in this regard cannot cease.

In conclusion, dormant tumor recurrence has important clinical implications that impact diagnosis, treatment, prognosis, and patient well-being. Continued research efforts aimed at understanding the mechanisms of tumor dormancy, the role of HSPs, and unending efforts to develop novel therapeutic approaches are essential for improving outcomes for patients with recurrent cancer.

## 7. Conclusions and Perspectives

The tumor and tumor cell dormancy and reactivation microenvironments have received enormous attention in the last decade. Still, many unanswered questions remain, particularly about the dynamics of tumor dormancy and reactivation in brain cancer. Through the repeated cycles of the dormant phase and reactivation in tumors, more dormant tumor cells migrate to secondary tissue microenvironments far from the primary tumor with diverse cellular heterogeneity and phenotypes that harbor distinct genetic signatures. These episodes are often mild, with no evident clinical symptomatology. Studies in tumor cell dormancy have exemplified the corroborative role of the HSP in promoting tumor and tumor cell dormancy, reactivation, and aggressive invasion following metastatic relapse. These hibernation and reactivation cycles may represent an evolutionary mechanism and genetically imprinted mutational strategy crucial to the dormant cancer cells’ survival and capacity to avert extinction.

In the future, efforts should be directed towards understanding the ECM components and HSP interactions that support tumor dormancy and the reactivation of dormant tumor cells. Insights into the biology of the dormant-promoting niche will inform the design and repurposing of targeted therapies. Likewise, insights into the dormancy niche can be exploited to restrict tumor growth from metastasizing into other tissues, which makes excision almost certainly impossible in some cases.

Additionally, knowledge of important HSP indicators that can accurately predict the propensity of dormant tumor cell development and reactivation would be crucial for planning prompt therapies. Also, another dimension of the cancer biology of the dormant tumor cell will be revealed by analyzing the mode and speed of migration post-reactivation. As these elements are regulated via specific signaling pathways, they present an opportunity for targeted treatment to pursue better patient outcomes.

## Abbreviations

CTCs	Circulating tumor cells	MKK4/7	Mitogen-activated protein kinase kinase 4/7
DTCs	Disseminated tumor cells	NR2F1	Orphan nuclear receptor
ERK	Extracellular regulatory kinase	TGF-β	Transforming growth factor beta
GBM	Glioblastoma multiforme	KISS1	KISS-1 metastasis suppressor
FGF	Fibroblast growth factor	CDKN1A	Cyclin-dependent Kinase 1A
HSP	Heat shock protein	Wnt	Wingless/integrated
MAPK	Mitogen-activated protein kinase	MAPK	Mitogen-activated protein kinase
MSK1	Mitogen and stress-activated kinase 1	TET	Ten–eleven translocation
mTOR	Mammalian target of rapamycin	HOXA9	Homeobox A9
TGF	Transforming growth factor	CD82	Cluster of differentiation 82 molecule
TME	Tumor microenvironment	AKAP12	A-kinase-anchoring protein 12
MMP	Matrix Metalloproteinases	FoxM1	Forkhead box M1
JNK	C-Jun N-terminal kinase	RPS6KB1	Ribosomal protein S6 Kinase B1
TBX2	T-box transcription factor-2	EphA5	EPH ceceptor A5
*MAPKAPK2*	MAPK-activated protein kinase 2	IGFBP5	Insulin-like growth factor-binding protein 5
BHLHE41	Basic helix–loop–helix family member E41	H2BK	H2B clustered histone 12
HSPA5	Heat shock protein family A member 5	SOX9	SRY-box transcription factor 9
DDIT3	DNA damage-inducible transcript 3	RARβ	Retinoic acid receptor beta
NANOG	Nanog homeobox	miR	MicroRNA
DmiR	Dormancy-associated microRNA	CDK	Cyclin-dependent kinase
ATF7	Activating transcription factor 7	CKI	Cyclin-dependent kinase inhibitor
HSPA1B	Heat shock protein family A member 1B	Cip/Kip	Cyclin-dependent kinase inhibitor 1A
ESCC	Esophageal squamous cell carcinoma	uPAR	Plasminogen activator, urokinase receptor
HSF2	Heat shock transcription factor 2	uPA	Plasminogen activator, urokinase
GSK3β	Glycogen synthase kinase 3 beta	CXCR4	C-X-C motif chemokine receptor 4
DKK3	Dickkopf WNT-signaling pathway inhibitor 3	DDR1	Discoidin domain receptor tyrosine kinase 1
INK4	Cyclin-dependent kinase inhibitor 2A	Raf	Raf-1 proto-oncogen
HIF	Hypoxia-inducible factor	TSP-1	Th-1rombospondin
AXL	AXL receptor tyrosine kinase	ESM1	Endothelial cell-specific molecule 1
EGFR	Epithelial growth factor receptor	PI3K	Phosphoinositide 3-kinases
CTL	Cytotoxic T cells	IDO	Indolamine 2,3-dioxygenase
VEGF	Vascular endothelial growth factor	TNF-β	Tumor necrosis factor beta
GLUT1	Glucose transporter 1	AMPK	Adenosine monophosphate-activated protein kinase
GPD1	Glycerol-3-phosphate dehydrogenase 1	BCL-2	B-cell lymphoma 2
BECN1	Beclin 1	Atg	Autophagy related gene
PFKFB3	Phosphofructo-2-kinase/Fructose-2,6-Bisphosphate 3	ARHI	Aplasia Ras homolog member 1
LC3	Microtubule-associated protein light chain 3	PRKAB1	p53-activated 5’-AMP-activated protein kinase subunit beta-1
CMA	Chaperone-mediated autophagy	LAMP2A	Lysosomal associated membrane protein 2A
MRN	MRN Complex interacting protein	ChK1	Cell cycle checkpoint kinase 1
PP2A	Protein phosphatase 2	CIP2A	Cellular Inhibitor of PP2A
HRE	Hypoxia response elements	CTLR	C-type lectin receptor
SR	Scavenger receptor	COX2	Cyclooxygenase 2
BRAF	B-Raf Proto-oncogen	MEK	Mitogen-activated protein kinase kinase 1
ERBB2/HER2	Erb-B2 receptor tyrosine kinase 2	TERT	Telomerase reverse transcriptase
AKT	Protein kinase B	TP53	Tumor protein p53
VHL	Von Hippel–Lindau tumor suppressor	DNAJA3	DnaJ heat shock protein family member A3
SREC-1	Scavenger receptor expressed by endothelial cells-1	DRAM1	Damage-regulated autophagy modulator-1
TLR	Toll-like receptor	IFN-γ	Interferon gamma
MDSC	Myeloid-derived suppressor cells	ADAMTs	A disintegrin and metalloproteinases with thrombospondin motifs
UTR	Untranslated region	SRC	Src proto-oncogen
LOX	Lysine oxidase	GRP	Glucose-regulated protein
NET	Neutrophil extracellular traps	tPA	Tissue-type plasminogen
EMT	Epithelial-to-mesenchymal transition	LRP1	Low-density lipoprotein receptor-related protein 1
GSC	Glioma stem cell	ITGB1	Integrin subunit beta 1
LAMC1	Laminin subunit gamma 1	PD-1	Programmed cell death protein 1
FAK	Focal adhesion kinase	LMD	Leptomeningeal dissemination
PTPRZ1	Protein tyrosine phosphatase receptor	PLEKHA7	Pleckstrin homolog domain-containing A7
CTC	Circulating tumor cell		

## References

[1] Pe’er D., Ogawa S., Elhanani O., Keren L., Oliver T.G., Wedge D. (2021). Tumor heterogeneity. Cancer Cell.

[2] Siddika A., Chowdhury S., Hasan M.R., Moniruzzaman M., Been Sayeed S.K.J., Tabassum T., Chowduary M., Tabassum T., Islam A., Rahman M.M. (2023). Clinicopathological Patterns of Malignant Solid Tumors in Adult Patients: A Hospital-Based Study From Bangladesh. Cureus.

[3] Balayan V., Guddati A.K. (2022). Tumor Dormancy: Biologic and Therapeutic Implications. World J. Oncol..

[4] Phan T.G., Croucher P.I. (2020). The dormant cancer cell life cycle. Nat. Rev. Cancer.

[5] Hu C., Yang J., Qi Z., Wu H., Wang B., Zou F., Mei H., Liu J., Wang W., Liu Q. (2022). Heat shock proteins: Biological functions, pathological roles, and therapeutic opportunities. MedComm (2020).

[6] Yoneda A., Minomi K., Tamura Y. (2021). Heat shock protein 47 confers chemoresistance on pancreatic cancer cells by interacting with calreticulin and IRE1α. Cancer Sci..

[7] Gehrmann M., Specht H.M., Bayer C., Brandstetter M., Chizzali B., Duma M., Breuninger S., Hube K., Lehnerer S., van Phi V. (2014). Hsp70—A biomarker for tumor detection and monitoring of outcome of radiation therapy in patients with squamous cell carcinoma of the head and neck. Radiat. Oncol..

[8] Xiao X., Wang W., Li Y., Yang D., Li X., Shen C., Liu Y., Ke X., Guo S., Guo Z. (2018). HSP90AA1-mediated autophagy promotes drug resistance in osteosarcoma. J. Exp. Clin. Cancer Res..

[9] Albakova Z., Armeev G.A., Kanevskiy L.M., Kovalenko E.I., Sapozhnikov A.M. (2020). HSP70 Multi-Functionality in Cancer. Cells.

[10] Wang Y., Chen Q., Wu D., Chen Q., Gong G., He L., Wu X. (2021). Lamin-A interacting protein Hsp90 is required for DNA damage repair and chemoresistance of ovarian cancer cells. Cell Death Dis..

[11] Ling X., Wan J., Peng B., Chen J. (2021). Hsp70 Promotes SUMO of HIF-1α and Promotes Lung Cancer Invasion and Metastasis. J. Oncol..

[12] Nagaraju G.P., Long T.E., Park W., Landry J.C., Taliaferro-Smith L., Farris A.B., Diaz R., El-Rayes B.F. (2015). Heat shock protein 90 promotes epithelial to mesenchymal transition, invasion, and migration in colorectal cancer. Mol. Carcinog..

[13] Vahid S., Thaper D., Gibson K.F., Bishop J.L., Zoubeidi A. (2016). Molecular chaperone Hsp27 regulates the Hippo tumor suppressor pathway in cancer. Sci. Rep..

[14] Sung H., Ferlay J., Siegel R.L., Laversanne M., Soerjomataram I., Jemal A., Bray F. (2021). Global Cancer Statistics 2020: GLOBOCAN Estimates of Incidence and Mortality Worldwide for 36 Cancers in 185 Countries. CA Cancer J. Clin..

[15] Siegel R.L., Giaquinto A.N., Jemal A. (2024). Cancer statistics, 2024. CA Cancer J. Clin..

[16] Okano H., Temple S. (2009). Cell types to order: Temporal specification of CNS stem cells. Curr. Opin. Neurobiol..

[17] Min H.-Y., Lee H.-Y. (2023). Cellular Dormancy in Cancer: Mechanisms and Potential Targeting Strategies. Cancer Res. Treat..

[18] Marescal O., Cheeseman I.M. (2020). Cellular Mechanisms and Regulation of Quiescence. Dev. Cell.

[19] Agudo J., Park E.S., Rose S.A., Alibo E., Sweeney R., Dhainaut M., Kobayashi K.S., Sachidanandam R., Baccarini A., Merad M. (2018). Quiescent Tissue Stem Cells Evade Immune Surveillance. Immunity.

[20] Hen O., Barkan D. (2020). Dormant disseminated tumor cells and cancer stem/progenitor-like cells: Similarities and opportunities. Semin. Cancer Biol..

[21] Bragado P., Estrada Y., Parikh F., Krause S., Capobianco C., Farina H.G., Schewe D.M., Aguirre-Ghiso J.A. (2013). TGF-β2 dictates disseminated tumour cell fate in target organs through TGF-β-RIII and p38α/β signalling. Nat. Cell Biol..

[22] Gawrzak S., Rinaldi L., Gregorio S., Arenas E.J., Salvador F., Urosevic J., Figueras-Puig C., Rojo F., del Barco Barrantes I., Cejalvo J.M. (2018). MSK1 regulates luminal cell differentiation and metastatic dormancy in ER+ breast cancer. Nat. Cell Biol..

[23] Liu S., Dontu G., Mantle I.D., Patel S., Ahn N.-s., Jackson K.W., Suri P., Wicha M.S. (2006). Hedgehog Signaling and Bmi-1 Regulate Self-renewal of Normal and Malignant Human Mammary Stem Cells. Cancer Res..

[24] Kobayashi A., Okuda H., Xing F., Pandey P.R., Watabe M., Hirota S., Pai S.K., Liu W., Fukuda K., Chambers C. (2011). Bone morphogenetic protein 7 in dormancy and metastasis of prostate cancer stem-like cells in bone. J. Exp. Med..

[25] Gillen A.E., Riemondy K.A., Amani V., Griesinger A.M., Gilani A., Venkataraman S., Madhavan K., Prince E., Sanford B., Hankinson T.C. (2020). Single-Cell RNA Sequencing of Childhood Ependymoma Reveals Neoplastic Cell Subpopulations That Impact Molecular Classification and Etiology. Cell Rep..

[26] Aguirre-Ghiso J.A., Estrada Y., Liu D., Ossowski L. (2003). ERK(MAPK) activity as a determinant of tumor growth and dormancy; regulation by p38(SAPK). Cancer Res..

[27] Prunier C., Alay A., van Dijk M., Ammerlaan K.L., van Gelderen S., Marvin D.L., Teunisse A., Slieker R.C., Szuhai K., Jochemsen A.G. (2021). Breast cancer dormancy is associated with a 4NG1 state and not senescence. npj Breast Cancer.

[28] Wu M.-Z., Chen S.-F., Nieh S., Benner C., Ger L.-P., Jan C.-I., Ma L., Chen C.-H., Hishida T., Chang H.-T. (2015). Hypoxia Drives Breast Tumor Malignancy through a TET–TNFα–p38–MAPK Signaling Axis. Cancer Res..

[29] Sun M., Song C.-X., Huang H., Frankenberger C.A., Sankarasharma D., Gomes S., Chen P., Chen J., Chada K.K., He C. (2013). HMGA2/TET1/HOXA9 signaling pathway regulates breast cancer growth and metastasis. Proc. Natl. Acad. Sci. USA.

[30] Chrysanthou S., Tang Q., Lee J., Taylor S.J., Zhao Y., Steidl U., Zheng D., Dawlaty M.M. (2022). The DNA dioxygenase Tet1 regulates H3K27 modification and embryonic stem cell biology independent of its catalytic activity. Nucleic Acids Res..

[31] Hsu C.-H., Peng K.-L., Kang M.-L., Chen Y.-R., Yang Y.-C., Tsai C.-H., Chu C.-S., Jeng Y.-M., Chen Y.-T., Lin F.-M. (2012). TET1 Suppresses Cancer Invasion by Activating the Tissue Inhibitors of Metalloproteinases. Cell Rep..

[32] Steeg P.S. (2003). Metastasis suppressors alter the signal transduction of cancer cells. Nat. Rev. Cancer.

[33] Lee J.-W., Hur J., Kwon Y.-W., Chae C.-W., Choi J.-I., Hwang I., Yun J.-Y., Kang J.-A., Choi Y.-E., Kim Y.H. (2021). KAI1(CD82) is a key molecule to control angiogenesis and switch angiogenic milieu to quiescent state. J. Hematol. Oncol..

[34] Vander Griend D.J., Kocherginsky M., Hickson J.A., Stadler W.M., Lin A., Rinker-Schaeffer C.W. (2005). Suppression of Metastatic Colonization by the Context-Dependent Activation of the c-Jun NH2-Terminal Kinase Kinases JNKK1/MKK4 and MKK7. Cancer Res..

[35] Lefter L.P., Sunamura M., Furukawa T., Takeda K., Kotobuki N., Oshimura M., Matsuno S., Horii A. (2003). Inserting Chromosome 18 into Pancreatic Cancer Cells Switches Them to a Dormant Metastatic Phenotype. Clin. Cancer Res..

[36] Suzuki E., Ota T., Tsukuda K., Okita A., Matsuoka K., Murakami M., Doihara H., Shimizu N. (2004). nm23-H1 reduces in vitro cell migration and the liver metastatic potential of colon cancer cells by regulating myosin light chain phosphorylation. Int. J. Cancer.

[37] Che G., Chen J., Liu L., Wang Y., Li L., Qin Y., Zhou Q. (2006). Transfection of nm23-H1 increased expression of beta-Catenin, E-Cadherin and TIMP-1 and decreased the expression of MMP-2, CD44v6 and VEGF and inhibited the metastatic potential of human non-small cell lung cancer cell line L9981. Neoplasma.

[38] Corno C., Perego P. (2019). KiSS1 in regulation of metastasis and response to antitumor drugs. Drug Resist. Updates.

[39] Prince S., Carreira S., Vance K.W., Abrahams A., Goding C.R. (2004). Tbx2 Directly Represses the Expression of the p21WAF1 Cyclin-Dependent Kinase Inhibitor. Cancer Res..

[40] Li K., Wu X., Li Y., Hu T.-T., Wang W., Gonzalez F.J., Liu W. (2023). AKAP12 promotes cancer stem cell-like phenotypes and activates STAT3 in colorectal cancer. Clin. Transl. Oncol..

[41] Yamada S.D., Hickson J.A., Hrobowski Y., Vander Griend D.J., Benson D., Montag A., Karrison T., Huo D., Rutgers J., Adams S. (2002). Mitogen-activated protein kinase kinase 4 (MKK4) acts as a metastasis suppressor gene in human ovarian carcinoma. Cancer Res..

[42] Lotan T., Hickson J., Souris J., Huo D., Taylor J., Li T., Otto K., Yamada S.D., Macleod K., Rinker-Schaeffer C.W. (2008). c-Jun NH2-Terminal Kinase Activating Kinase 1/Mitogen-Activated Protein Kinase Kinase 4–Mediated Inhibition of SKOV3ip.1 Ovarian Cancer Metastasis Involves Growth Arrest and p21 Up-regulation. Cancer Res..

[43] Azmi S., Ozog A., Taneja R. (2004). Sharp-1/DEC2 Inhibits Skeletal Muscle Differentiation through Repression of Myogenic Transcription Factors*. J. Biol. Chem..

[44] Ferrer-Martínez A., Marotta M., Baldán A., Haro D., Gómez-Foix A.M. (2004). Chicken ovalbumin upstream promoter-transcription factor I represses the transcriptional activity of the human muscle glycogen phosphorylase promoter in C2C12 cells. Biochim. Et Biophys. Acta (BBA) Gene Struct. Expr..

[45] Adamski V., Hempelmann A., Flüh C., Lucius R., Synowitz M., Hattermann K., Held-Feindt J. (2017). Dormant glioblastoma cells acquire stem cell characteristics and are differentially affected by Temozolomide and AT101 treatment. Oncotarget.

[46] Adam A.P., George A., Schewe D., Bragado P., Iglesias B.V., Ranganathan A.C., Kourtidis A., Conklin D.S., Aguirre-Ghiso J.A. (2009). Computational Identification of a p38SAPK-Regulated Transcription Factor Network Required for Tumor Cell Quiescence. Cancer Res..

[47] Stepniak E., Ricci R., Eferl R., Sumara G., Sumara I., Rath M., Hui L., Wagner E.F. (2006). c-Jun/AP-1 controls liver regeneration by repressing p53/p21 and p38 MAPK activity. Genes Dev..

[48] Sosa M.S., Parikh F., Maia A.G., Estrada Y., Bosch A., Bragado P., Ekpin E., George A., Zheng Y., Lam H.-M. (2015). NR2F1 controls tumour cell dormancy via SOX9- and RARβ-driven quiescence programmes. Nature Commun..

[49] Liu H., Sun Q., Sun Y., Zhang J., Yuan H., Pang S., Qi X., Wang H., Zhang M., Zhang H. (2017). MELK and EZH2 Cooperate to Regulate Medulloblastoma Cancer Stem-like Cell Proliferation and Differentiation. Mol. Cancer Res..

[50] Wang J., Qi Q., Zhou W., Feng Z., Huang B., Chen A., Zhang D., Li W., Zhang Q., Jiang Z. (2018). Inhibition of glioma growth by flavokawain B is mediated through endoplasmic reticulum stress induced autophagy. Autophagy.

[51] Hermann A., Gowher H., Jeltsch A. (2004). Biochemistry and biology of mammalian DNA methyltransferases. Cell. Mol. Life Sci. CMLS.

[52] Gaudet F., Hodgson J.G., Eden A., Jackson-Grusby L., Dausman J., Gray J.W., Leonhardt H., Jaenisch R. (2003). Induction of Tumors in Mice by Genomic Hypomethylation. Science.

[53] Tiram G., Segal E., Krivitsky A., Shreberk-Hassidim R., Ferber S., Ofek P., Udagawa T., Edry L., Shomron N., Roniger M. (2016). Identification of Dormancy-Associated MicroRNAs for the Design of Osteosarcoma-Targeted Dendritic Polyglycerol Nanopolyplexes. ACS Nano.

[54] Almog N., Ma L., Schwager C., Brinkmann B.G., Beheshti A., Vajkoczy P., Folkman J., Hlatky L., Abdollahi A. (2012). Consensus Micro RNAs Governing the Switch of Dormant Tumors to the Fast-Growing Angiogenic Phenotype. PLoS ONE.

[55] Song F., Wei M., Wang J., Liu Y., Guo M., Li X., Luo J., Zhou J., Wang M., Guo D. (2019). Hepatitis B virus-regulated growth of liver cancer cells occurs through the microRNA-340-5p-activating transcription factor 7-heat shock protein A member 1B axis. Cancer Sci..

[56] Gao H., Chakraborty G., Lee-Lim A.P., Mavrakis K.J., Wendel H.-G., Giancotti F.G. (2014). Forward genetic screens in mice uncover mediators and suppressors of metastatic reactivation. Proc. Natl. Acad. Sci. USA.

[57] Meng X., Chen X., Lu P., Ma W., Yue D., Song L., Fan Q. (2017). miR-202 Promotes Cell Apoptosis in Esophageal Squamous Cell Carcinoma by Targeting HSF2. Oncol. Res..

[58] Weston W.A., Barr A.R. (2023). A cell cycle centric view of tumour dormancy. Br. J. Cancer.

[59] Garcia-Martinez L., Zhang Y., Nakata Y., Chan H.L., Morey L. (2021). Epigenetic mechanisms in breast cancer therapy and resistance. Nat. Commun..

[60] Fuchs E., Sahai E., Weeraratna A.T., Deneen B., Chak-Lui Wong C., Simon A. (2023). Understanding the microenvironment and how this controls cell fate. Dev. Cell.

[61] Butturini E., Carcereri de Prati A., Boriero D., Mariotto S. (2019). Tumor Dormancy and Interplay with Hypoxic Tumor Microenvironment. Int. J. Mol. Sci..

[62] Pietras K., Östman A. (2010). Hallmarks of cancer: Interactions with the tumor stroma. Exp. Cell Res..

[63] Brassart-Pasco S., Brézillon S., Brassart B., Ramont L., Oudart J.-B., Monboisse J.C. (2020). Tumor Microenvironment: Extracellular Matrix Alterations Influence Tumor Progression. Front. Oncol..

[64] Di Martino J.S., Akhter T., Bravo-Cordero J.J. (2021). Remodeling the ECM: Implications for Metastasis and Tumor Dormancy. Cancers.

[65] Endo H., Inoue M. (2019). Dormancy in cancer. Cancer Sci..

[66] Akman M., Belisario D.C., Salaroglio I.C., Kopecka J., Donadelli M., De Smaele E., Riganti C. (2021). Hypoxia, endoplasmic reticulum stress and chemoresistance: Dangerous liaisons. J. Exp. Clin. Cancer Res..

[67] Aqbi H.F., Wallace M., Sappal S., Payne K.K., Manjili M.H. (2018). IFN-γ orchestrates tumor elimination, tumor dormancy, tumor escape, and progression. J. Leukoc. Biol..

[68] Barkan D., Kleinman H., Simmons J.L., Asmussen H., Kamaraju A.K., Hoenorhoff M.J., Liu Z.-y., Costes S.V., Cho E.H., Lockett S. (2008). Inhibition of Metastatic Outgrowth from Single Dormant Tumor Cells by Targeting the Cytoskeleton. Cancer Res..

[69] Barkan D., Green J.E., Chambers A.F. (2010). Extracellular matrix: A gatekeeper in the transition from dormancy to metastatic growth. Eur. J. Cancer (Oxf. Engl. 1990).

[70] Mukherjee A., Bravo-Cordero J.J. (2023). Regulation of dormancy during tumor dissemination: The role of the ECM. Cancer Metastasis Rev..

[71] Gomis R.R., Gawrzak S. (2017). Tumor cell dormancy. Mol. Oncol..

[72] Tong L., Yi L., Liu P., Abeysekera I.R., Hai L., Li T., Tao Z., Ma H., Xie Y., Huang Y. (2018). Tumour cell dormancy as a contributor to the reduced survival of GBM patients who received standard therapy. Oncol. Rep..

[73] Correa R.J.M., Peart T., Valdes Y.R., DiMattia G.E., Shepherd T.G. (2011). Modulation of AKT activity is associated with reversible dormancy in ascites-derived epithelial ovarian cancer spheroids. Carcinogenesis.

[74] Keeratichamroen S., Lirdprapamongkol K., Svasti J. (2018). Mechanism of ECM-induced dormancy and chemoresistance in A549 human lung carcinoma cells. Oncol. Rep..

[75] Di Martino J.S., Nobre A.R., Mondal C., Taha I., Farias E.F., Fertig E.J., Naba A., Aguirre-Ghiso J.A., Bravo-Cordero J.J. (2022). A tumor-derived type III collagen-rich ECM niche regulates tumor cell dormancy. Nat. Cancer.

[76] Khoonkari M., Liang D., Kamperman M., Kruyt F.A.E., van Rijn P. (2022). Physics of Brain Cancer: Multiscale Alterations of Glioblastoma Cells under Extracellular Matrix Stiffening. Pharmaceutics.

[77] Beliveau A., Thomas G., Gong J., Wen Q., Jain A. (2016). Aligned Nanotopography Promotes a Migratory State in Glioblastoma Multiforme Tumor Cells. Sci. Rep..

[78] Carmeliet P., Jain R.K. (2011). Principles and mechanisms of vessel normalization for cancer and other angiogenic diseases. Nat. Rev. Drug Discov..

[79] Chen C.-W., Yang C.-H., Lin Y.-H., Hou Y.-C., Cheng T.-J., Chang S.-T., Huang Y.-H., Chung S.-T., Chio C.-C., Shan Y.-S. (2021). The Fibronectin Expression Determines the Distinct Progressions of Malignant Gliomas via Transforming Growth Factor-Beta Pathway. Int. J. Mol. Sci..

[80] Farino Reyes C.J., Pradhan S., Slater J.H. (2021). The Influence of Ligand Density and Degradability on Hydrogel Induced Breast Cancer Dormancy and Reactivation. Adv. Healthc. Mater..

[81] Ferreira L.P., Gaspar V.M., Mano J.F. (2020). Decellularized Extracellular Matrix for Bioengineering Physiomimetic 3D in Vitro Tumor Models. Trends Biotechnol..

[82] De Bock K., Cauwenberghs S., Carmeliet P. (2011). Vessel abnormalization: Another hallmark of cancer? Molecular mechanisms and therapeutic implications. Curr. Opin. Genet. Dev..

[83] Potente M., Gerhardt H., Carmeliet P. (2011). Basic and therapeutic aspects of angiogenesis. Cell.

[84] LaMonte G., Tang X., Chen J.L.-Y., Wu J., Ding C.-K.C., Keenan M.M., Sangokoya C., Kung H.-N., Ilkayeva O., Boros L.G. (2013). Acidosis induces reprogramming of cellular metabolism to mitigate oxidative stress. Cancer Metab..

[85] Gatenby R.A., Gillies R.J. (2004). Why do cancers have high aerobic glycolysis?. Nat. Rev. Cancer.

[86] Balgi A.D., Diering G.H., Donohue E., Lam K.K.Y., Fonseca B.D., Zimmerman C., Numata M., Roberge M. (2011). Regulation of mTORC1 Signaling by pH. PLoS ONE.

[87] Aguirre-Ghiso J.A., Ossowski L., Rosenbaum S.K. (2004). Green Fluorescent Protein Tagging of Extracellular Signal-Regulated Kinase and p38 Pathways Reveals Novel Dynamics of Pathway Activation during Primary and Metastatic Growth. Cancer Res..

[88] Hjelmeland A.B., Wu Q., Heddleston J.M., Choudhary G.S., MacSwords J., Lathia J.D., McLendon R., Lindner D., Sloan A., Rich J.N. (2011). Acidic stress promotes a glioma stem cell phenotype. Cell Death Differ..

[89] Goretzki L., Schmitt M., Mann K., Calvete J., Chucholowski N., Kramer M., Günzler W.A., Jänicke F., Graeff H. (1992). Effective activation of the proenzyme form of the urokinase-type plasminogen activator (pro-uPA) by the cysteine protease cathepsin L.. FEBS Lett..

[90] Gatenby R.A., Gawlinski E.T., Gmitro A.F., Kaylor B., Gillies R.J. (2006). Acid-mediated tumor invasion: A multidisciplinary study. Cancer Res..

[91] Shiozawa Y., Pedersen E.A., Patel L.R., Ziegler A.M., Havens A.M., Jung Y., Wang J., Zalucha S., Loberg R.D., Pienta K.J. (2010). GAS6/AXL Axis Regulates Prostate Cancer Invasion, Proliferation, and Survival in the Bone Marrow Niche. Neoplasia.

[92] Ghajar C.M., Peinado H., Mori H., Matei I.R., Evason K.J., Brazier H., Almeida D., Koller A., Hajjar K.A., Stainier D.Y.R. (2013). The perivascular niche regulates breast tumour dormancy. Nat. Cell Biol..

[93] Gattazzo F., Urciuolo A., Bonaldo P. (2014). Extracellular matrix: A dynamic microenvironment for stem cell niche. Biochim. Biophys. Acta (BBA)—Gen. Subj..

[94] Kerever A., Schnack J., Vellinga D., Ichikawa N., Moon C., Arikawa-Hirasawa E., Efird J.T., Mercier F. (2009). Novel Extracellular Matrix Structures in the Neural Stem Cell Niche Capture the Neurogenic Factor Fibroblast Growth Factor 2 from the Extracellular Milieu. Stem Cells.

[95] Tamamouna V., Pavlou E., Neophytou C.M., Papageorgis P., Costeas P. (2022). Regulation of Metastatic Tumor Dormancy and Emerging Opportunities for Therapeutic Intervention. Int. J. Mol. Sci..

[96] Chang Y., Chen J. (2021). Dormant mechanisms reveal the clinical significance of tumor dormancy: A narrative review. Ann. Blood.

[97] Satchi-Fainaro R., Ferber S., Segal E., Ma L., Dixit N., Ijaz A., Hlatky L., Abdollahi A., Almog N. (2012). Prospective Identification of Glioblastoma Cells Generating Dormant Tumors. PLoS ONE.

[98] Almog N., Ma L., Raychowdhury R., Schwager C., Erber R., Short S., Hlatky L., Vajkoczy P., Huber P.E., Folkman J. (2009). Transcriptional Switch of Dormant Tumors to Fast-Growing Angiogenic Phenotype. Cancer Res..

[99] Indraccolo S., Stievano L., Minuzzo S., Tosello V., Esposito G., Piovan E., Zamarchi R., Chieco-Bianchi L., Amadori A. (2006). Interruption of tumor dormancy by a transient angiogenic burst within the tumor microenvironment. Proc. Natl. Acad. Sci. USA.

[100] Giuriato S., Ryeom S., Fan A.C., Bachireddy P., Lynch R.C., Rioth M.J., van Riggelen J., Kopelman A.M., Passegué E., Tang F. (2006). Sustained regression of tumors upon MYC inactivation requires p53 or thrombospondin-1 to reverse the angiogenic switch. Proc. Natl. Acad. Sci. USA.

[101] Mohiuddin E., Wakimoto H. (2021). Extracellular matrix in glioblastoma: Opportunities for emerging therapeutic approaches. Am. J. Cancer Res..

[102] Winkler J., Abisoye-Ogunniyan A., Metcalf K.J., Werb Z. (2020). Concepts of extracellular matrix remodelling in tumour progression and metastasis. Nat. Commun..

[103] Schreiber R.D., Old L.J., Smyth M.J. (2011). Cancer immunoediting: Integrating immunity’s roles in cancer suppression and promotion. Science.

[104] Wang D., Anderson J.C., Gladson C.L. (2005). The Role of the Extracellular Matrix in Angiogenesis in Malignant Glioma Tumors. Brain Pathol..

[105] Zamarron B.F., Chen W. (2011). Dual Roles of Immune Cells and Their Factors in Cancer Development and Progression. Int. J. Biol. Sci..

[106] Tsai C.-H., Chuang Y.-M., Li X., Yu Y.-R., Tzeng S.-F., Teoh S.T., Lindblad K.E., Di Matteo M., Cheng W.-C., Hsueh P.-C. (2023). Immunoediting instructs tumor metabolic reprogramming to support immune evasion. Cell Metab..

[107] Hou X., Chen S., Zhang P., Guo D., Wang B. (2022). Targeted Arginine Metabolism Therapy: A Dilemma in Glioma Treatment. Front. Oncol..

[108] Rackaityte E., Halkias J. (2020). Mechanisms of Fetal T Cell Tolerance and Immune Regulation. Front. Immunol..

[109] Sandén E., Enríquez Pérez J., Visse E., Kool M., Carén H., Siesjö P., Darabi A. (2016). Preoperative systemic levels of VEGFA, IL-7, IL-17A, and TNF-β delineate two distinct groups of children with brain tumors. Pediatr. Blood Cancer.

[110] Sampson J.H., Gunn M.D., Fecci P.E., Ashley D.M. (2020). Brain immunology and immunotherapy in brain tumours. Nat. Rev. Cancer.

[111] Abedalthagafi M., Mobark N., Al-Rashed M., AlHarbi M. (2021). Epigenomics and immunotherapeutic advances in pediatric brain tumors. npj Precis. Oncol..

[112] Hosseinalizadeh H., Mahmoodpour M., Samadani A.A., Roudkenar M.H. (2022). The immunosuppressive role of indoleamine 2, 3-dioxygenase in glioblastoma: Mechanism of action and immunotherapeutic strategies. Med. Oncol..

[113] Parisi R., Patel R.R., Rood G., Bowden A., Turco G., Korones D.N., Andolina J.R., Comito M., Barth M., Weintraub L. (2023). Multi-institution analysis of tumor mutational burden and outcomes in pediatric central nervous system tumor patients. Pediatr. Blood Cancer.

[114] Patel R.R., Ramkissoon S.H., Ross J., Weintraub L. (2020). Tumor mutational burden and driver mutations: Characterizing the genomic landscape of pediatric brain tumors. Pediatr. Blood Cancer.

[115] Ranganathan A.C., Zhang L., Adam A.P., Aguirre-Ghiso J.A. (2006). Functional Coupling of p38-Induced Up-regulation of BiP and Activation of RNA-Dependent Protein Kinase–Like Endoplasmic Reticulum Kinase to Drug Resistance of Dormant Carcinoma Cells. Cancer Res..

[116] Mohrin M., Bourke E., Alexander D., Warr M.R., Barry-Holson K., Le Beau M.M., Morrison C.G., Passegué E. (2010). Hematopoietic Stem Cell Quiescence Promotes Error-Prone DNA Repair and Mutagenesis. Cell Stem Cell.

[117] Magnus N., D’Asti E., Meehan B., Garnier D., Rak J. (2014). Oncogenes and the coagulation system–forces that modulate dormant and aggressive states in cancer. Thromb. Res..

[118] Nobre A.R., Entenberg D., Wang Y., Condeelis J., Aguirre-Ghiso J.A. (2018). The Different Routes to Metastasis via Hypoxia-Regulated Programs. Trends Cell Biol..

[119] Fluegen G., Avivar-Valderas A., Wang Y., Padgen M.R., Williams J.K., Nobre A.R., Calvo V., Cheung J.F., Bravo-Cordero J.J., Entenberg D. (2017). Phenotypic heterogeneity of disseminated tumour cells is preset by primary tumour hypoxic microenvironments. Nat. Cell Biol..

[120] Muz B., de la Puente P., Azab F., Azab A.K. (2015). The role of hypoxia in cancer progression, angiogenesis, metastasis, and resistance to therapy. Hypoxia.

[121] Bardag-Gorce F., Hoffman C., Meepe I., Ferrini M., Hoft R.H., Oliva J., Niihara Y. (2023). Thrombospondin-1 induction and VEGF reduction by proteasome inhibition. Heliyon.

[122] Zanotelli M.R., Reinhart-King C.A., Dong C., Zahir N., Konstantopoulos K. (2018). Mechanical Forces in Tumor Angiogenesis. Biomechanics in Oncology.

[123] Harris A.L. (2002). Hypoxi—A key regulatory factor in tumour growth. Nat. Rev. Cancer.

[124] Bayko L., Rak J., Man S., Bicknell R., Ferrara N., Kerbel R.S. (1998). The dormant in vivo phenotype of early stage primary human melanoma: Termination by overexpression of vascular endothelial growth factor. Angiogenesis.

[125] Holmgren L., O’Reilly M.S., Folkman J. (1995). Dormancy of micrometastases: Balanced proliferation and apoptosis in the presence of angiogenesis suppression. Nat. Med..

[126] Johnson R.W., Finger E.C., Olcina M.M., Vilalta M., Aguilera T., Miao Y., Merkel A.R., Johnson J.R., Sterling J.A., Wu J.Y. (2016). Induction of LIFR confers a dormancy phenotype in breast cancer cells disseminated to the bone marrow. Nat. Cell Biol..

[127] Icard P., Fournel L., Wu Z., Alifano M., Lincet H. (2019). Interconnection between Metabolism and Cell Cycle in Cancer. Trends Biochem. Sci..

[128] Yuan H.-X., Xiong Y., Guan K.-L. (2013). Nutrient Sensing, Metabolism, and Cell Growth Control. Mol. Cell.

[129] Viale A., Pettazzoni P., Lyssiotis C.A., Ying H., Sánchez N., Marchesini M., Carugo A., Green T., Seth S., Giuliani V. (2014). Oncogene ablation-resistant pancreatic cancer cells depend on mitochondrial function. Nature.

[130] Viale A., Corti D., Draetta G.F. (2015). Tumors and mitochondrial respiration: A neglected connection. Cancer Res..

[131] Hampsch R.A., Wells J.D., Traphagen N.A., McCleery C.F., Fields J.L., Shee K., Dillon L.M., Pooler D.B., Lewis L.D., Demidenko E. (2020). AMPK Activation by Metformin Promotes Survival of Dormant ER(+) Breast Cancer Cells. Clin. Cancer Res..

[132] Rehman G., Shehzad A., Khan A.L., Hamayun M. (2014). Role of AMP-activated protein kinase in cancer therapy. Arch. Pharm..

[133] Vlashi E., Lagadec C., Vergnes L., Matsutani T., Masui K., Poulou M., Popescu R., Della Donna L., Evers P., Dekmezian C. (2011). Metabolic state of glioma stem cells and nontumorigenic cells. Proc. Natl. Acad. Sci. USA.

[134] Ciavardelli D., Rossi C., Barcaroli D., Volpe S., Consalvo A., Zucchelli M., De Cola A., Scavo E., Carollo R., D’Agostino D. (2014). Breast cancer stem cells rely on fermentative glycolysis and are sensitive to 2-deoxyglucose treatment. Cell Death Dis..

[135] Lagadinou E.D., Sach A., Callahan K., Rossi R.M., Neering S.J., Minhajuddin M., Ashton J.M., Pei S., Grose V., O’Dwyer K.M. (2013). BCL-2 inhibition targets oxidative phosphorylation and selectively eradicates quiescent human leukemia stem cells. Cell Stem Cell.

[136] Lin S., Li K., Qi L. (2023). Cancer stem cells in brain tumors: From origin to clinical implications. MedComm.

[137] Rusu P., Shao C., Neuerburg A., Acikgöz A.A., Wu Y., Zou P., Phapale P., Shankar T.S., Döring K., Dettling S. (2019). GPD1 Specifically Marks Dormant Glioma Stem Cells with a Distinct Metabolic Profile. Cell Stem Cell.

[138] Pepe-Mooney B.J., Dill M.T., Alemany A., Ordovas-Montanes J., Matsushita Y., Rao A., Sen A., Miyazaki M., Anakk S., Dawson P.A. (2019). Single-Cell Analysis of the Liver Epithelium Reveals Dynamic Heterogeneity and an Essential Role for YAP in Homeostasis and Regeneration. Cell Stem Cell.

[139] Vera-Ramirez L., Vodnala S.K., Nini R., Hunter K.W., Green J.E. (2018). Autophagy promotes the survival of dormant breast cancer cells and metastatic tumour recurrence. Nat. Commun..

[140] Straume O., Shimamura T., Lampa M.J., Carretero J., Øyan A.M., Jia D., Borgman C.L., Soucheray M., Downing S.R., Short S.M. (2012). Suppression of heat shock protein 27 induces long-term dormancy in human breast cancer. Proc. Natl. Acad. Sci. USA.

[141] Csizmadia T., Juhász G., Martinez A.B., Galluzzi L. (2020). Chapter Eleven—Crinophagy mechanisms and its potential role in human health and disease. Progress in Molecular Biology and Translational Science.

[142] Baba M., Takeshige K., Baba N., Ohsumi Y. (1994). Ultrastructural analysis of the autophagic process in yeast: Detection of autophagosomes and their characterization. J. Cell Biol..

[143] Yue Z., Jin S., Yang C., Levine A.J., Heintz N. (2003). Beclin 1, an autophagy gene essential for early embryonic development, is a haploinsufficient tumor suppressor. Proc. Natl. Acad. Sci. USA.

[144] Takamura A., Komatsu M., Hara T., Sakamoto A., Kishi C., Waguri S., Eishi Y., Hino O., Tanaka K., Mizushima N. (2011). Autophagy-deficient mice develop multiple liver tumors. Genes Dev..

[145] La Belle Flynn A., Calhoun B.C., Sharma A., Chang J.C., Almasan A., Schiemann W.P. (2019). Autophagy inhibition elicits emergence from metastatic dormancy by inducing and stabilizing Pfkfb3 expression. Nat. Commun..

[146] Lu Z., Luo R.Z., Lu Y., Zhang X., Yu Q., Khare S., Kondo S., Kondo Y., Yu Y., Mills G.B. (2008). The tumor suppressor gene ARHI regulates autophagy and tumor dormancy in human ovarian cancer cells. J. Clin. Investig..

[147] Tasdemir E., Maiuri M.C., Galluzzi L., Vitale I., Djavaheri-Mergny M., D’Amelio M., Criollo A., Morselli E., Zhu C., Harper F. (2008). Regulation of autophagy by cytoplasmic p53. Nat. Cell Biol..

[148] Kenzelmann Broz D., Spano Mello S., Bieging K.T., Jiang D., Dusek R.L., Brady C.A., Sidow A., Attardi L.D. (2013). Global genomic profiling reveals an extensive p53-regulated autophagy program contributing to key p53 responses. Genes Dev..

[149] Crighton D., Wilkinson S., O’Prey J., Syed N., Smith P., Harrison P.R., Gasco M., Garrone O., Crook T., Ryan K.M. (2006). DRAM, a p53-Induced Modulator of Autophagy, Is Critical for Apoptosis. Cell.

[150] Yang Y., Karsli-Uzunbas G., Poillet-Perez L., Sawant A., Hu Z.S., Zhao Y., Moore D., Hu W., White E. (2020). Autophagy promotes mammalian survival by suppressing oxidative stress and p53. Genes Dev..

[151] Han Q., Deng Y., Chen S., Chen R., Yang M., Zhang Z., Sun X., Wang W., He Y., Wang F. (2017). Downregulation of ATG5-dependent macroautophagy by chaperone-mediated autophagy promotes breast cancer cell metastasis. Sci. Rep..

[152] Dong S., Wang Q., Kao Y.R., Diaz A., Tasset I., Kaushik S., Thiruthuvanathan V., Zintiridou A., Nieves E., Dzieciatkowska M. (2021). Chaperone-mediated autophagy sustains haematopoietic stem-cell function. Nature.

[153] Park C., Suh Y., Cuervo A.M. (2015). Regulated degradation of Chk1 by chaperone-mediated autophagy in response to DNA damage. Nat. Commun..

[154] García-Gutiérrez L., Delgado M.D., León J. (2019). MYC Oncogene Contributions to Release of Cell Cycle Brakes. Genes.

[155] Bretones G., Delgado M.D., León J. (2015). Myc and cell cycle control. Biochim. Biophys. Acta.

[156] Gomes L.R., Menck C.F.M., Cuervo A.M. (2017). Chaperone-mediated autophagy prevents cellular transformation by regulating MYC proteasomal degradation. Autophagy.

[157] Puustinen P., Jäättelä M. (2014). KIAA1524/CIP2A promotes cancer growth by coordinating the activities of MTORC1 and MYC. Autophagy.

[158] Rao S., Tortola L., Perlot T., Wirnsberger G., Novatchkova M., Nitsch R., Sykacek P., Frank L., Schramek D., Komnenovic V. (2014). A dual role for autophagy in a murine model of lung cancer. Nat. Commun..

[159] Rosenfeldt M.T., O’Prey J., Morton J.P., Nixon C., MacKay G., Mrowinska A., Au A., Rai T.S., Zheng L., Ridgway R. (2013). p53 status determines the role of autophagy in pancreatic tumour development. Nature.

[160] Yamamoto K., Venida A., Yano J., Biancur D.E., Kakiuchi M., Gupta S., Sohn A.S.W., Mukhopadhyay S., Lin E.Y., Parker S.J. (2020). Autophagy promotes immune evasion of pancreatic cancer by degrading MHC-I. Nature.

[161] Auzmendi-Iriarte J., Matheu A. (2022). Intrinsic role of chaperone-mediated autophagy in cancer stem cell maintenance. Autophagy.

[162] Auzmendi-Iriarte J., Otaegi-Ugartemendia M., Carrasco-Garcia E., Azkargorta M., Diaz A., Saenz-Antoñanzas A., Andermatten J.A., Garcia-Puga M., Garcia I., Elua-Pinin A. (2022). Chaperone-Mediated Autophagy Controls Proteomic and Transcriptomic Pathways to Maintain Glioma Stem Cell Activity. Cancer Res..

[163] Yun C.W., Lee S.H. (2018). The Roles of Autophagy in Cancer. Int. J. Mol. Sci..

[164] Dubrez L., Causse S., Borges Bonan N., Dumétier B., Garrido C. (2020). Heat-shock proteins: Chaperoning DNA repair. Oncogene.

[165] Kriegenburg F., Ellgaard L., Hartmann-Petersen R. (2012). Molecular chaperones in targeting misfolded proteins for ubiquitin-dependent degradation. FEBS J..

[166] Baird N.A., Turnbull D.W., Johnson E.A. (2006). Induction of the heat shock pathway during hypoxia requires regulation of heat shock factor by hypoxia-inducible factor-1. J. Biol. Chem..

[167] Sullivan E.K., Weirich C.S., Guyon J.R., Sif S., Kingston R.E. (2001). Transcriptional activation domains of human heat shock factor 1 recruit human SWI/SNF. Mol. Cell. Biol..

[168] Kijima T., Prince T.L., Tigue M.L., Yim K.H., Schwartz H., Beebe K., Lee S., Budzynski M.A., Williams H., Trepel J.B. (2018). HSP90 inhibitors disrupt a transient HSP90-HSF1 interaction and identify a noncanonical model of HSP90-mediated HSF1 regulation. Sci. Rep..

[169] Zou J., Guo Y., Guettouche T., Smith D.F., Voellmy R. (1998). Repression of heat shock transcription factor HSF1 activation by HSP90 (HSP90 complex) that forms a stress-sensitive complex with HSF1. Cell.

[170] Taha E.A., Ono K., Eguchi T. (2019). Roles of Extracellular HSPs as Biomarkers in Immune Surveillance and Immune Evasion. Int. J. Mol. Sci..

[171] Santagata S., Hu R., Lin N.U., Mendillo M.L., Collins L.C., Hankinson S.E., Schnitt S.J., Whitesell L., Tamimi R.M., Lindquist S. (2011). High levels of nuclear heat-shock factor 1 (HSF1) are associated with poor prognosis in breast cancer. Proc. Natl. Acad. Sci. USA.

[172] Trepel J., Mollapour M., Giaccone G., Neckers L. (2010). Targeting the dynamic HSP90 complex in cancer. Nat. Rev. Cancer.

[173] Babi A., Menlibayeva K., Bex T., Doskaliev A., Akshulakov S., Shevtsov M. (2022). Targeting Heat Shock Proteins in Malignant Brain Tumors: From Basic Research to Clinical Trials. Cancers.

[174] Graner M.W., Alzate O., Dechkovskaia A.M., Keene J.D., Sampson J.H., Mitchell D.A., Bigner D.D. (2009). Proteomic and immunologic analyses of brain tumor exosomes. FASEB J. Off. Publ. Fed. Am. Soc. Exp. Biol..

[175] Albakova Z., Siam M.K.S., Sacitharan P.K., Ziganshin R.H., Ryazantsev D.Y., Sapozhnikov A.M. (2021). Extracellular heat shock proteins and cancer: New perspectives. Transl. Oncol..

[176] Graner M.W., Cumming R.I., Bigner D.D. (2007). The Heat Shock Response and Chaperones/Heat Shock Proteins in Brain Tumors: Surface Expression, Release, and Possible Immune Consequences. J. Neurosci..

[177] Reddy V.S., Madala S.K., Trinath J., Reddy G.B. (2018). Extracellular small heat shock proteins: Exosomal biogenesis and function. Cell Stress Chaperones.

[178] Sojka D.R., Abramowicz A., Adamiec-Organiściok M., Karnas E., Mielańczyk Ł., Kania D., Blamek S., Telka E., Scieglinska D. (2023). Heat shock protein A2 is a novel extracellular vesicle-associated protein. Sci. Rep..

[179] Murshid A., Theriault J., Gong J., Calderwood S.K., Calderwood S.K., Prince T.L. (2011). Investigating Receptors for Extracellular Heat Shock Proteins. Molecular Chaperones: Methods and Protocols.

[180] Hanahan D., Folkman J. (1996). Patterns and Emerging Mechanisms of the Angiogenic Switch during Tumorigenesis. Cell.

[181] Jain R.K., di Tomaso E., Duda D.G., Loeffler J.S., Sorensen A.G., Batchelor T.T. (2007). Angiogenesis in brain tumours. Nat. Rev. Neurosci..

[182] Chatterjee S., Bhattacharya S., Socinski M.A., Burns T.F. (2016). HSP90 inhibitors in lung cancer: Promise still unfulfilled. Clin. Adv. Hematol. Oncol..

[183] WORKMAN P., BURROWS F., NECKERS L., ROSEN N. (2007). Drugging the Cancer Chaperone HSP90. Ann. New York Acad. Sci..

[184] Chiosis G., Vilenchik M., Kim J., Solit D. (2004). Hsp90: The vulnerable chaperone. Drug Discov. Today.

[185] Neckers L. (2006). Using Natural Product Inhibitors to Validate Hsp90 as a Molecular Target in Cancer. Curr. Top. Med. Chem..

[186] Hadchity E., Aloy M.-T., Paulin C., Armandy E., Watkin E., Rousson R., Gleave M., Chapet O., Rodriguez-Lafrasse C. (2009). Heat Shock Protein 27 as a New Therapeutic Target for Radiation Sensitization of Head and Neck Squamous Cell Carcinoma. Mol. Ther..

[187] Colvin T.A., Gabai V.L., Gong J., Calderwood S.K., Li H., Gummuluru S., Matchuk O.N., Smirnova S.G., Orlova N.V., Zamulaeva I.A. (2014). Hsp70–Bag3 Interactions Regulate Cancer-Related Signaling Networks. Cancer Res..

[188] Isaacs J.S., Jung Y.-J., Mimnaugh E.G., Martinez A., Cuttitta F., Neckers L.M. (2002). Hsp90 Regulates a von Hippel Lindau-independent Hypoxia-inducible Factor-1α-degradative Pathway. J. Biol. Chem..

[189] Jiang X., Zhou T., Wang Z., Qi B., Xia H. (2017). HSP47 Promotes Glioblastoma Stemlike Cell Survival by Modulating Tumor Microenvironment Extracellular Matrix through TGF-β Pathway. ACS Chem. Neurosci..

[190] Zhang Z., Jing J., Ye Y., Chen Z., Jing Y., Li S., Hong W., Ruan H., Liu Y., Hu Q. (2020). Characterization of the dual functional effects of heat shock proteins (HSPs) in cancer hallmarks to aid development of HSP inhibitors. Genome Med..

[191] Bae M.-K., Jeong J.-W., Kim S.-H., Kim S.-Y., Kang H.J., Kim D.-M., Bae S.-K., Yun I., Trentin G.A., Rozakis-Adcock M. (2005). Tid-1 Interacts with the von Hippel-Lindau Protein and Modulates Angiogenesis by Destabilization of HIF-1α. Cancer Res..

[192] Suto R., Srivastava P.K. (1995). A mechanism for the specific immunogenicity of heat shock protein-chaperoned peptides. Science.

[193] Bae J., Munshi A., Li C., Samur M., Prabhala R., Mitsiades C., Anderson K.C., Munshi N.C. (2013). Heat Shock Protein 90 Is Critical for Regulation of Phenotype and Functional Activity of Human T Lymphocytes and NK Cells. J. Immunol..

[194] Mittal D., Gubin M.M., Schreiber R.D., Smyth M.J. (2014). New insights into cancer immunoediting and its three component phases--elimination, equilibrium and escape. Curr. Opin. Immunol..

[195] Ngiow S.F., Teng M.W.L., Smyth M.J. (2013). A balance of interleukin-12 and -23 in cancer. Trends Immunol..

[196] Teng M.W., Vesely M.D., Duret H., McLaughlin N., Towne J.E., Schreiber R.D., Smyth M.J. (2012). Opposing roles for IL-23 and IL-12 in maintaining occult cancer in an equilibrium state. Cancer Res..

[197] Ravi V.M., Neidert N., Will P., Joseph K., Maier J.P., Kückelhaus J., Vollmer L., Goeldner J.M., Behringer S.P., Scherer F. (2022). T-cell dysfunction in the glioblastoma microenvironment is mediated by myeloid cells releasing interleukin-10. Nat. Commun..

[198] Anderson A.C., Joller N., Kuchroo V.K. (2016). Lag-3, Tim-3, and TIGIT: Co-inhibitory Receptors with Specialized Functions in Immune Regulation. Immunity.

[199] Wu X., Peng M., Huang B., Zhang H., Wang H., Huang B., Xue Z., Zhang L., Da Y., Yang D. (2013). Immune microenvironment profiles of tumor immune equilibrium and immune escape states of mouse sarcoma. Cancer Lett..

[200] Gehrmann M., Marienhagen J., Eichholtz-Wirth H., Fritz E., Ellwart J., Jäättelä M., Zilch T., Multhoff G. (2005). Dual function of membrane-bound heat shock protein 70 (Hsp70), Bag-4, and Hsp40: Protection against radiation-induced effects and target structure for natural killer cells. Cell Death Differ..

[201] Chalmin F., Ladoire S., Mignot G., Vincent J., Bruchard M., Remy-Martin J.P., Boireau W., Rouleau A., Simon B., Lanneau D. (2010). Membrane-associated Hsp72 from tumor-derived exosomes mediates STAT3-dependent immunosuppressive function of mouse and human myeloid-derived suppressor cells. J. Clin. Investig..

[202] Grabovska Y., Mackay A., O’Hare P., Crosier S., Finetti M., Schwalbe E.C., Pickles J.C., Fairchild A.R., Avery A., Cockle J. (2020). Pediatric pan-central nervous system tumor analysis of immune-cell infiltration identifies correlates of antitumor immunity. Nat. Commun..

[203] Gröbner S.N., Worst B.C., Weischenfeldt J., Buchhalter I., Kleinheinz K., Rudneva V.A., Johann P.D., Balasubramanian G.P., Segura-Wang M., Brabetz S. (2018). The landscape of genomic alterations across childhood cancers. Nature.

[204] Ruoslahti E. (1996). Brain extracellular matrix. Glycobiology.

[205] Mott J.D., Werb Z. (2004). Regulation of matrix biology by matrix metalloproteinases. Curr. Opin. Cell Biol..

[206] Cabral-Pacheco G.A., Garza-Veloz I., Castruita-De la Rosa C., Ramirez-Acuña J.M., Perez-Romero B.A., Guerrero-Rodriguez J.F., Martinez-Avila N., Martinez-Fierro M.L. (2020). The Roles of Matrix Metalloproteinases and Their Inhibitors in Human Diseases. Int. J. Mol. Sci..

[207] Sims J.D., McCready J., Jay D.G. (2011). Extracellular Heat Shock Protein (Hsp)70 and Hsp90α Assist in Matrix Metalloproteinase-2 Activation and Breast Cancer Cell Migration and Invasion. PLoS ONE.

[208] Correia A.L., Mori H., Chen E.I., Schmitt F.C., Bissell M.J. (2013). The hemopexin domain of MMP3 is responsible for mammary epithelial invasion and morphogenesis through extracellular interaction with HSP90β. Genes Dev..

[209] Blasi F., Carmeliet P. (2002). uPAR: A versatile signalling orchestrator. Nat. Rev. Mol. Cell Biol..

[210] Lin Y., Peng N., Zhuang H., Zhang D., Wang Y., Hua Z.-C. (2014). Heat shock proteins HSP70 and MRJ cooperatively regulate cell adhesion and migration through urokinase receptor. BMC Cancer.

[211] Zhang Y., Ghazwani M., Li J., Sun M., Stolz D.B., He F., Fan J., Xie W., Li S. (2014). MiR-29b inhibits collagen maturation in hepatic stellate cells through down-regulating the expression of HSP47 and lysyl oxidase. Biochem. Biophys. Res. Commun..

[212] Cox T.R., Erler J.T. (2011). Remodeling and homeostasis of the extracellular matrix: Implications for fibrotic diseases and cancer. Dis. Model. Mech..

[213] Nobuhisa T., Naomoto Y., Okawa T., Takaoka M., Gunduz M., Motoki T., Nagatsuka H., Tsujigiwa H., Shirakawa Y., Yamatsuji T. (2007). Translocation of heparanase into nucleus results in cell differentiation. Cancer Sci..

[214] Koo B.H., Apte S.S. (2010). Cell-surface processing of the metalloprotease pro-ADAMTS9 is influenced by the chaperone GRP94/gp96. J. Biol. Chem..

[215] Koo B.-H., Longpré J.-M., Somerville R.P.T., Alexander J.P., Leduc R., Apte S.S. (2007). Regulation of ADAMTS9 Secretion and Enzymatic Activity by Its Propeptide*. J. Biol. Chem..

[216] Martin-Rufino J.D., Castano N., Pang M., Grody E.I., Joubran S., Caulier A., Wahlster L., Li T., Qiu X., Riera-Escandell A.M. (2023). Massively parallel base editing to map variant effects in human hematopoiesis. Cell.

[217] Wang T., Rodina A., Dunphy M.P., Corben A., Modi S., Guzman M.L., Gewirth D.T., Chiosis G. (2019). Chaperome heterogeneity and its implications for cancer study and treatment. J. Biol. Chem..

[218] Bolaender A., Zatorska D., He H., Joshi S., Sharma S., Digwal C.S., Patel H.J., Sun W., Imber B.S., Ochiana S.O. (2021). Chemical tools for epichaperome-mediated interactome dysfunctions of the central nervous system. Nat. Commun..

[219] Rodina A., Wang T., Yan P., Gomes E.D., Dunphy M.P.S., Pillarsetty N., Koren J., Gerecitano J.F., Taldone T., Zong H. (2016). The epichaperome is an integrated chaperome network that facilitates tumour survival. Nature.

[220] Zylicz M., King F.W., Wawrzynow A. (2001). Hsp70 interactions with the p53 tumour suppressor protein. EMBO J..

[221] Blagosklonny M.V., Toretsky J., Bohen S., Neckers L. (1996). Mutant conformation of p53 translated in vitro or in vivo requires functional HSP90. Proc. Natl. Acad. Sci. USA.

[222] Bruey J.-M., Ducasse C., Bonniaud P., Ravagnan L., Susin S.A., Diaz-Latoud C., Gurbuxani S., Arrigo A.-P., Kroemer G., Solary E. (2000). Hsp27 negatively regulates cell death by interacting with cytochrome c. Nat. Cell Biol..

[223] Stetler R.A., Gao Y., Signore A.P., Cao G., Chen J. (2009). HSP27: Mechanisms of cellular protection against neuronal injury. Curr. Mol. Med..

[224] Toogun O.A., Dezwaan D.C., Freeman B.C. (2008). The hsp90 molecular chaperone modulates multiple telomerase activities. Mol. Cell. Biol..

[225] DeZwaan D.C., Freeman B.C. (2010). HSP90 manages the ends. Trends Biochem. Sci..

[226] Chaklader M., Das P., Pereira J.A., Law A., Chattopadhyay S., Chatterjee R., Mondal A., Law S. (2012). 17-AAG mediated targeting of Hsp90 limits tert activity in peritoneal sarcoma related malignant ascites by downregulating cyclin D1 during cell cycle entry. Exp. Oncol..

[227] O’Callaghan-Sunol C., Gabai V.L., Sherman M.Y. (2007). Hsp27 Modulates p53 Signaling and Suppresses Cellular Senescence. Cancer Res..

[228] Murphy M.E. (2013). The HSP70 family and cancer. Carcinogenesis.

[229] Sha G., Jiang Z., Zhang W., Jiang C., Wang D., Tang D. (2023). The multifunction of HSP70 in cancer: Guardian or traitor to the survival of tumor cells and the next potential therapeutic target. Int. Immunopharmacol..

[230] Zhang J., Li H., Liu Y., Zhao K., Wei S., Sugarman E.T., Liu L., Zhang G. (2022). Targeting HSP90 as a Novel Therapy for Cancer: Mechanistic Insights and Translational Relevance. Cells.

[231] Salam R., Saliou A., Bielle F., Bertrand M., Antoniewski C., Carpentier C., Alentorn A., Capelle L., Sanson M., Huillard E. (2023). Cellular senescence in malignant cells promotes tumor progression in mouse and patient Glioblastoma. Nat. Commun..

[232] Chen J., Li Y., Yu T.-S., McKay R.M., Burns D.K., Kernie S.G., Parada L.F. (2012). A restricted cell population propagates glioblastoma growth after chemotherapy. Nature.

[233] Pradhan S., Sperduto J.L., Farino C.J., Slater J.H. (2018). Engineered In Vitro Models of Tumor Dormancy and Reactivation. J. Biol. Eng..

[234] Krall J.A., Reinhardt F., Mercury O.A., Pattabiraman D.R., Brooks M.W., Dougan M., Lambert A.W., Bierie B., Ploegh H.L., Dougan S.K. (2018). The systemic response to surgery triggers the outgrowth of distant immune-controlled tumors in mouse models of dormancy. Sci. Transl. Med..

[235] Albrengues J., Shields M.A., Ng D., Park C.G., Ambrico A., Poindexter M.E., Upadhyay P., Uyeminami D.L., Pommier A., Küttner V. (2018). Neutrophil extracellular traps produced during inflammation awaken dormant cancer cells in mice. Science.

[236] Poggio P., Sorge M., Seclì L., Brancaccio M. (2021). Extracellular HSP90 Machineries Build Tumor Microenvironment and Boost Cancer Progression. Front. Cell Dev. Biol..

[237] Gopal U., Bohonowych J.E., Lema-Tome C., Liu A., Garrett-Mayer E., Wang B., Isaacs J.S. (2011). A Novel Extracellular Hsp90 Mediated Co-Receptor Function for LRP1 Regulates EphA2 Dependent Glioblastoma Cell Invasion. PLoS ONE.

[238] Tian Y., Wang C., Chen S., Liu J., Fu Y., Luo Y. (2019). Extracellular Hsp90α and clusterin synergistically promote breast cancer epithelial-to-mesenchymal transition and metastasis via LRP1. J. Cell Sci..

[239] Rajesh Y., Banerjee A., Pal I., Biswas A., Das S., Dey K.K., Kapoor N., Ghosh A.K., Mitra P., Mandal M. (2019). Delineation of crosstalk between HSP27 and MMP-2/MMP-9: A synergistic therapeutic avenue for glioblastoma management. Biochim. Biophys. Acta (BBA)—Gen. Subj..

[240] Thuringer D., Jego G., Wettstein G., Terrier O., Cronier L., Yousfi N., Hébrard S., Bouchot A., Hazoumé A., Joly A.-L. (2013). Extracellular HSP27 mediates angiogenesis through Toll-like receptor 3. FASEB J..

[241] Chaiwatanasirikul K.A., Sala A. (2011). The tumour-suppressive function of CLU is explained by its localisation and interaction with HSP60. Cell Death Dis..

[242] Zhao D., Jiang X., Yao C., Zhang L., Liu H., Xia H., Wang Y. (2014). Heat shock protein 47 regulated by miR-29a to enhance glioma tumor growth and invasion. J. Neuro-Oncol..

[243] Jin H.O., Hong S.E., Kim J.Y., Kim M.R., Chang Y.H., Hong Y.J., Lee J.K., Park I.C. (2019). Induction of HSP27 and HSP70 by constitutive overexpression of Redd1 confers resistance of lung cancer cells to ionizing radiation. Oncol. Rep..

[244] Brondani Da Rocha A., Regner A., Grivicich I., Pretto Schunemann D., Diel C., Kovaleski G., Brunetto De Farias C., Mondadori E., Almeida L., Braga Filho A. (2004). Radioresistance is associated to increased Hsp70 content in human glioblastoma cell lines. Int. J. Oncol..

[245] Rouard N., Peiffert D., Rio E., Mahé M.A., Delpon G., Marchesi V., Falk A.T., Salleron J., Serre A.A. (2019). Intensity-modulated radiation therapy of anal squamous cell carcinoma: Relationship between delineation quality and regional recurrence. Radiother. Oncol..

[246] Chang L., Ni J., Beretov J., Wasinger V.C., Hao J., Bucci J., Malouf D., Gillatt D., Graham P.H., Li Y. (2017). Identification of protein biomarkers and signaling pathways associated with prostate cancer radioresistance using label-free LC-MS/MS proteomic approach. Sci. Rep..

[247] McLaughlin M., Barker H.E., Khan A.A., Pedersen M., Dillon M., Mansfield D.C., Patel R., Kyula J.N., Bhide S.A., Newbold K.L. (2017). HSP90 inhibition sensitizes head and neck cancer to platin-based chemoradiotherapy by modulation of the DNA damage response resulting in chromosomal fragmentation. BMC Cancer.

[248] Li H.K., Matsumoto Y., Furusawa Y., Kamada T. (2016). PU-H71, a novel Hsp90 inhibitor, as a potential cancer-specific sensitizer to carbon-ion beam therapy. J. Radiat. Res..

[249] Lianos G.D., Alexiou G.A., Mangano A., Mangano A., Rausei S., Boni L., Dionigi G., Roukos D.H. (2015). The role of heat shock proteins in cancer. Cancer Lett..

[250] Morita R., Nishizawa S., Torigoe T., Takahashi A., Tamura Y., Tsukahara T., Kanaseki T., Sokolovskaya A., Kochin V., Kondo T. (2014). Heat shock protein DNAJB8 is a novel target for immunotherapy of colon cancer-initiating cells. Cancer Sci..

[251] Gehrmann M., Stangl S., Kirschner A., Foulds G.A., Sievert W., Doß B.T., Walch A., Pockley A.G., Multhoff G. (2012). Immunotherapeutic Targeting of Membrane Hsp70-Expressing Tumors Using Recombinant Human Granzyme B. PLoS ONE.

[252] Mbofung R.M., McKenzie J.A., Malu S., Zhang M., Peng W., Liu C., Kuiatse I., Tieu T., Williams L., Devi S. (2017). HSP90 inhibition enhances cancer immunotherapy by upregulating interferon response genes. Nat. Commun..

[253] Alexiou G.A., Vartholomatos G., Stefanaki K., Patereli A., Dova L., Karamoutsios A., Lallas G., Sfakianos G., Moschovi M., Prodromou N. (2013). Expression of heat shock proteins in medulloblastoma. J. Neurosurg. Pediatr..

[254] Bruschi M., Petretto A., Cama A., Pavanello M., Bartolucci M., Morana G., Ramenghi L.A., Garré M.L., Ghiggeri G.M., Panfoli I. (2021). Potential biomarkers of childhood brain tumor identified by proteomics of cerebrospinal fluid from extraventricular drainage (EVD). Sci. Rep..

[255] Sun G., Cao Y., Xu Y., Huai D., Chen P., Guo J., Li M., Dai Y. (2019). Overexpression of Hsc70 promotes proliferation, migration, and invasion of human glioma cells. J. Cell. Biochem..

[256] Dafforn T.R., Della M., Miller A.D. (2001). The Molecular Interactions of Heat Shock Protein 47 (Hsp47) and Their Implications for Collagen Biosynthesis. J. Biol. Chem..

[257] Zou M., Bhatia A., Dong H., Jayaprakash P., Guo J., Sahu D., Hou Y., Tsen F., Tong C., O’Brien K. (2017). Evolutionarily conserved dual lysine motif determines the non-chaperone function of secreted Hsp90alpha in tumour progression. Oncogene.

[258] Chou T.-Y., Chen W.-C., Lee A.-C., Hung S.-M., Shih N.-Y., Chen M.-Y. (2009). Clusterin silencing in human lung adenocarcinoma cells induces a mesenchymal-to-epithelial transition through modulating the ERK/Slug pathway. Cell. Signal..

[259] Chen F., Chandrashekar D.S., Scheurer M.E., Varambally S., Creighton C.J. (2022). Global molecular alterations involving recurrence or progression of pediatric brain tumors. Neoplasia.

[260] Masucci M.T., Minopoli M., Di Carluccio G., Motti M.L., Carriero M.V. (2022). Therapeutic Strategies Targeting Urokinase and Its Receptor in Cancer. Cancers.

[261] Aguirre-Ghiso J.A., Liu D., Mignatti A., Kovalski K., Ossowski L. (2001). Urokinase Receptor and Fibronectin Regulate the ERKMAPK to p38MAPK Activity Ratios That Determine Carcinoma Cell Proliferation or Dormancy In Vivo. Mol. Biol. Cell.

[262] Ghiso J.A.A., Kovalski K., Ossowski L. (1999). Tumor Dormancy Induced by Downregulation of Urokinase Receptor in Human Carcinoma Involves Integrin and MAPK Signaling. J. Cell Biol..

[263] Liu X., Chen J.Y., Chien Y., Yang Y.P., Chen M.T., Lin L.T. (2021). Overview of the molecular mechanisms of migration and invasion in glioblastoma multiforme. J. Chin. Med. Assoc. JCMA.

[264] Kwon H., Yun M., Kwon T.-H., Bang M., Lee J., Lee Y.S., Ko H.Y., Chong K. (2023). Fibronectin Type III Domain Containing 3B as a Potential Prognostic and Therapeutic Biomarker for Glioblastoma. Biomedicines.

[265] Kabir F., Apu M.N.H. (2022). Multi-omics analysis predicts fibronectin 1 as a prognostic biomarker in glioblastoma multiforme. Genomics.

[266] Trombetta-Lima M., Rosa-Fernandes L., Angeli C.B., Moretti I.F., Franco Y.M., Mousessian A.S., Wakamatsu A., Lerario A.M., Oba-Shinjo S.M., Pasqualucci C.A. (2021). Extracellular Matrix Proteome Remodeling in Human Glioblastoma and Medulloblastoma. J. Proteome Res..

[267] Jones J.K., Zhang H., Lyne A.-M., Cavalli F.M.G., Hassen W.E., Stevenson K., Kornahrens R., Yang Y., Li S., Dell S. (2023). ABL1 and ABL2 promote medulloblastoma leptomeningeal dissemination. Neuro-Oncol. Adv..

[268] Wang X., Sun Q., Wang W., Liu B., Gu Y., Chen L. (2023). Decoding key cell sub-populations and molecular alterations in glioblastoma at recurrence by single-cell analysis. Acta Neuropathol. Commun..

[269] Varn F.S., Johnson K.C., Martinek J., Huse J.T., Nasrallah M.P., Wesseling P., Cooper L.A.D., Malta T.M., Wade T.E., Sabedot T.S. (2022). Glioma progression is shaped by genetic evolution and microenvironment interactions. Cell.

[270] Wade A., Robinson A.E., Engler J.R., Petritsch C., James C.D., Phillips J.J. (2013). Proteoglycans and their roles in brain cancer. FEBS J..

[271] Barnes J.M., Kaushik S., Bainer R.O., Sa J.K., Woods E.C., Kai F., Przybyla L., Lee M., Lee H.W., Tung J.C. (2018). A tension-mediated glycocalyx–integrin feedback loop promotes mesenchymal-like glioblastoma. Nat. Cell Biol..

[272] Broders-Bondon F., Nguyen Ho-Bouldoires T.H., Fernandez-Sanchez M.-E., Farge E. (2018). Mechanotransduction in tumor progression: The dark side of the force. J. Cell Biol..

[273] Marozzi M., Parnigoni A., Negri A., Viola M., Vigetti D., Passi A., Karousou E., Rizzi F. (2021). Inflammation, Extracellular Matrix Remodeling, and Proteostasis in Tumor Microenvironment. Int. J. Mol. Sci..

[274] Acharekar A., Bachal K., Shirke P., Thorat R., Banerjee A., Gardi N., Majumder A., Dutt S. (2023). Substrate stiffness regulates the recurrent glioblastoma cell morphology and aggressiveness. Matrix Biol..

[275] Miroshnikova Y.A., Mouw J.K., Barnes J.M., Pickup M.W., Lakins J.N., Kim Y., Lobo K., Persson A.I., Reis G.F., McKnight T.R. (2016). Tissue mechanics promote IDH1-dependent HIF1α–tenascin C feedback to regulate glioblastoma aggression. Nat. Cell Biol..

[276] Davies C., Pan H., Godwin J., Gray R., Arriagada R., Raina V., Abraham M., Medeiros Alencar V.H., Badran A., Bonfill X. (2013). Long-term effects of continuing adjuvant tamoxifen to 10 years versus stopping at 5 years after diagnosis of oestrogen receptor-positive breast cancer: ATLAS, a randomised trial. Lancet.

[277] Meng F., Zhao Q., Zhao X., Yang C., Liu R., Pang J., Zhao W., Wang Q., Liu M., Zhang Z. (2022). A rice protein modulates endoplasmic reticulum homeostasis and coordinates with a transcription factor to initiate blast disease resistance. Cell Rep..

[278] Francescangeli F., De Angelis M.L., Rossi R., Cuccu A., Giuliani A., De Maria R., Zeuner A. (2023). Dormancy, stemness, and therapy resistance: Interconnected players in cancer evolution. Cancer Metastasis Rev..

[279] Dai Y., Wang L., Tang J., Cao P., Luo Z., Sun J., Kiflu A., Sai B., Zhang M., Wang F. (2016). Activation of anaphase-promoting complex by p53 induces a state of dormancy in cancer cells against chemotherapeutic stress. Oncotarget.

[280] Recasens A., Munoz L. (2019). Targeting Cancer Cell Dormancy. Trends Pharmacol. Sci..

[281] Wikman H., Vessella R., Pantel K. (2008). Cancer micrometastasis and tumour dormancy. APMIS.

[282] Rehman S.K., Haynes J., Collignon E., Brown K.R., Wang Y., Nixon A.M.L., Bruce J.P., Wintersinger J.A., Singh Mer A., Lo E.B.L. (2021). Colorectal Cancer Cells Enter a Diapause-like DTP State to Survive Chemotherapy. Cell.

